# School-based self-management interventions for asthma among primary school children: a systematic review

**DOI:** 10.1038/s41533-021-00230-2

**Published:** 2021-04-01

**Authors:** Siti Nurkamilla Ramdzan, Julia Suhaimi, Katherine M. Harris, Ee Ming Khoo, Su May Liew, Steve Cunningham, Hilary Pinnock

**Affiliations:** 1 Department of Primary Care Medicine, Faculty of Medicine, University of Malaya, Kuala Lumpur, Malaysia; 2 NIHR Global Health Research Unit on Respiratory Health (RESPIRE), Usher Institute, University of Edinburgh, Edinburgh, UK; 3 Centre for Child Health, Blizard Institute, Queen Mary University of London, London, UK

**Keywords:** Asthma, Paediatrics

## Abstract

A Cochrane review of school-based asthma interventions (combining all ages) found improved health outcomes. Self-management skills, however, vary according to age. We assessed effectiveness of primary school-based self-management interventions and identified components associated with successful programmes in children aged 6–12 years. We updated the Cochrane search (March 2020) and included the Global Health database. Two reviewers screened, assessed risk-of-bias and extracted data. We included 23 studies (10,682 participants); four at low risk-of-bias. Twelve studies reported at least one positive result for an outcome of interest. All 12 positive studies reported parental involvement in the intervention, compared to two-thirds of ineffective studies. In 10 of the 12 positive studies, parental involvement was substantial (e.g. attending sessions; phone/video communication) rather than being provided with written information. School-based self-management intervention can improve health outcomes and substantial parental involvement in school-based programmes seemed important for positive outcomes among primary school children.

## Introduction

Asthma, the commonest long-term condition among children, causes significant morbidity and mortality globally^1^. Asthma guidelines recommend supported self-management to improve asthma control and reduce the use of urgent healthcare services^2–4^. Supported self-management, which includes discussion about self-management and provision of a personalised asthma action plan supported by regular asthma review, can be delivered effectively in diverse cultural and demographic groups^5,6^.

School-based asthma self-management interventions have been reported to improve asthma control and reduce school absenteeism and asthma exacerbations^7–11^. However, most systematic reviews analysed combined data from primary and secondary schools (5–18 years)^7–10^. One scoping review conducted in 2014 focused on primary school children, but the aim was to identify research gaps rather than assess outcomes^11^. The Cochrane review (Harris, 2019) used meta-analyses to assess intervention effectiveness and qualitative comparative analysis to examine the components of successful implementations^7^. The authors identified a number of components as being important: theoretical underpinning, parental involvement, child satisfaction and conducting the intervention during lesson time. However, the Cochrane review included interventions directed at children and adolescents (5–18 years), and did not distinguish the components associated with effective interventions in primary school children, which may differ from adolescents^7^. Educational intervention needs to be age-appropriate as primary school children will have less autonomy and capability to self-manage asthma compared to adolescents^12^. Thus, we aimed to review the effectiveness of school-based self-management interventions for primary school children with asthma and to examine the components associated with successful programmes.

## Results

**Fig. 1:**
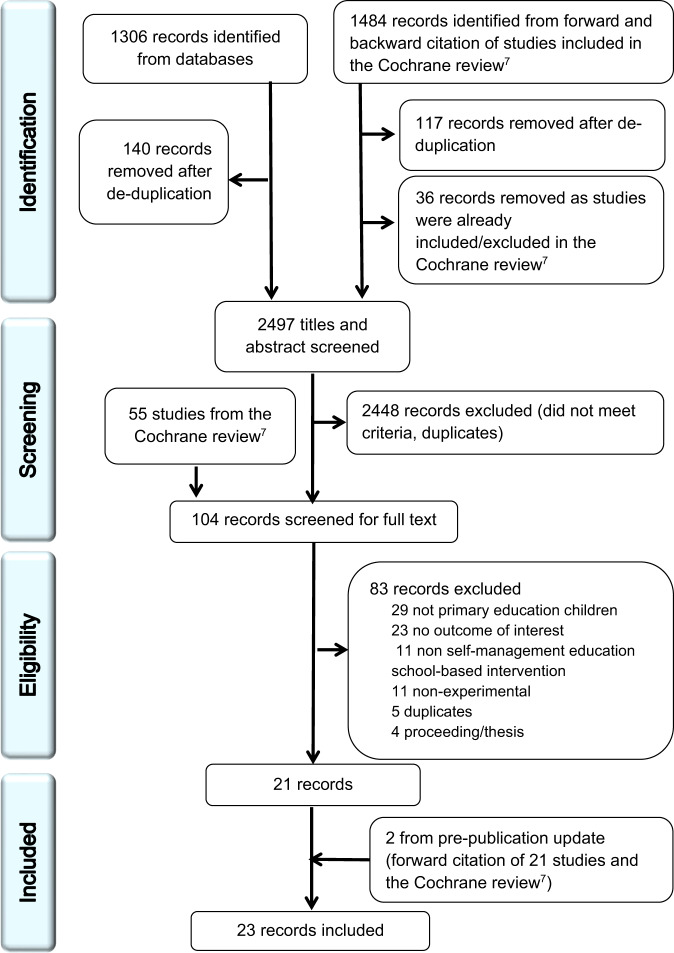
PRISMA diagram. This figure illustrates the article selection process using the PRISMA diagram.

Figure 1 illustrates the article selection process using the PRISMA diagram. We included 23 studies; 16 studies from the Cochrane review^13–28^, five studies from the updated database search^29–33^ and two studies from the pre-publication update^34,35^. The total number of participants was 10,682. Some studies did not report numbers in each group so we cannot provide number by allocation^13,14,24^. We contacted all authors for information not reported in the papers, and nine (39%) responded^13,14,25,27,29,30,32–34^.

### Characteristics of included studies

The interventions were conducted from 1992 to 2019. Seventeen studies were randomised controlled trials (RCTs) (14 cluster RCT^13–15,18–26,31,32^, three individual RCTs^27,29,34^), three were non-randomised studies^28,30,35^ and three were uncontrolled pre-and-post studies^16,17,33^. Fifteen studies were conducted in the United States^15–18,21,23–25,27–31,34,35^, four in Canada^13,14,19,20^, one each in Spain^32^ and United Kingdom^26^, and two in low- and middle- income countries (China and Thailand)^22,33^. All but one^17^ of the studies in the United States were conducted in minority populations^15,16,18,21,23–25,27–31,34,35^, two Canadian studies were conducted in majority population^19,20^; none of the others^13,14,22,26,32,33^ reported ethnicity of population studied.

### Overall intervention characteristics

The programmes were used to deliver self-management intervention varied. Eight studies used standard programmes (Open Airway for School (OAS) or tailored OAS^15,17,21–24,28,29^, four studies used Roaring Adventures of Puff (RAP) or tailored RAP^13,14,19,20^, and the other studies developed novel interventions^16,18,25–27,29–35^. The programmes ranged from one to eight sessions, and all were delivered by healthcare personnel, (school nurse, asthma educator, community nurse, respiratory therapist, physician) except for two that were delivered by trained school teachers^22,32^. Fifteen studies delivered the intervention in group sessions^13–17,19–26,28,34^, four used individual face-to-face sessions^27,29,30,35^, one used individual computer-assisted programme^18^ and another used individual telemedicine sessions^31^. Two studies were unclear^23,33^.

### Risk of bias in the included studies

**Fig. 2:**
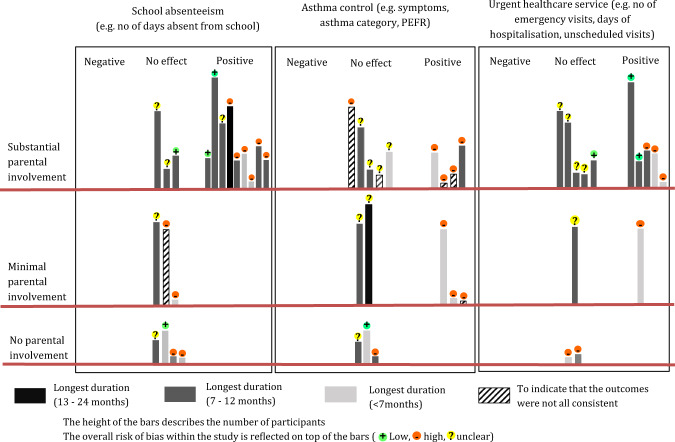
Harvest plot illustrating effectiveness of included studies across parental involvement. Harvest plot illustrating the effectiveness on school absenteeism, asthma control and urgent healthcare services across parental involvement for school-based self-management asthma educational intervention. The shading of the bars indicates the duration of the study and the height of the bars describes the number of participants. The overall risk of bias is reflected on top of the bars.

The overall RoB is given in summary Supplementary Table 1 (first column) and illustrated in the Harvest plot (Fig. 2). Details of the RoB are in Supplementary Table 2. Four studies had low overall risk of bias^13,14,20,32^, eleven had high risk of bias^15–17,21,27–30,33–35^ and eight were unclear^18,19,22–26,31^. Only seven (30%) studies were categorised at low risk in random sequence generation^13,14,20,21,23,32,34^. All uncontrolled studies were categorised as high-risk in four domains (random sequence generation, allocation concealment, baseline outcome similar and baseline character similar)^16,17,33^.

### Effectiveness of interventions

The effect of the interventions on each outcome of interest is detailed in Supplementary Table 1, with an explanation of how the direction of the effect was interpreted and the overall effect of the study assessed. Twelve studies (two at low risk-of-bias) were assessed as having an overall positive (beneficial) effect^13–17,21,22,29,30,33–35^ and eleven studies (two at low risk-of-bias) as having no effect^18–20,23–28,31,32^. No study was categorised as harmful or mixed effect. The Harvest plot (Fig. 2) illustrates the effect of varying degrees of parental involvement on school absenteeism, asthma control and urgent healthcare use.

### Study components according to CFIR sub-domains

The CIFR domains addressed in the studies are summarised in column 2 in Supplementary Table 1. Cicutto et al.^13^ was the only study that explicitly addressed all the CFIR sub-domains in their intervention; in contrast, Spencer et al.^17^ addressed only two sub-domains. All included studies used and measured the impact of at least one specific component in their intervention, e.g. information provision assessed as improvement of knowledge and self-management behaviour. The other commonly addressed sub-domain was parental involvement (19/23)^13–25,29–31,33–35^, though this varied in intensity (We use the term ‘parents’ to describe parents, guardians or other care-givers). See Supplementary Table 3 for definitions of involvement. Some studies had substantial involvement e.g. parents attending session or actively involved in phone/video communication^13–15,17,19–22,24,25,29–31,33,35^, while others had minimal parental involvement e.g. passive information in a letter^16,18,23,29,34^. Ten studies used theory to guide the development of the interventions; six used social cognitive theory^13,14,18–20,22^, two used Orem self-care theory^28,34^, one used life stress model^29^, and another was guided by Bruhn’s theoretical model^25^. Nine studies considered access to healthcare of their study population^13–15,24,25,27,30,32,33^.

### Association of CFIR sub-domains and effectiveness

Tables 1 and 2 are summary matrices comparing use of the 12 CFIR sub-domains in studies with overall positive or no effect (See Supplementary Table 4 for more detail). The number of CFIR sub-domains used varied widely (2 to 12) and was similar in the studies with positive/no effect.

**Table 1:**
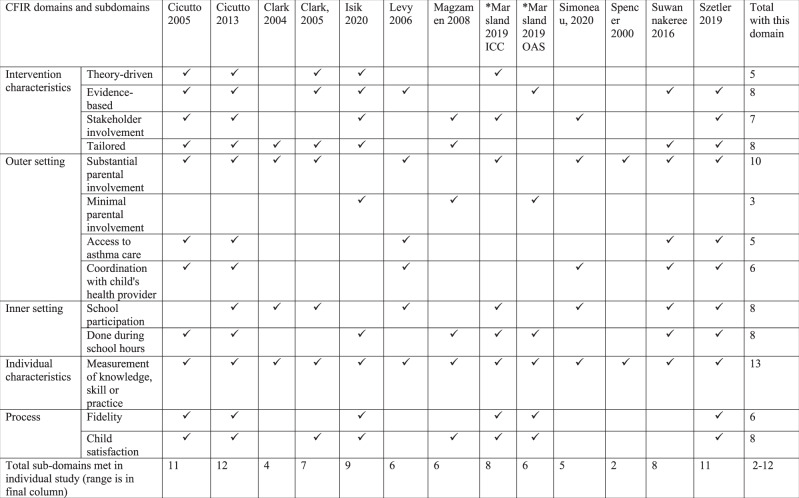
Summary matrix comparing 12 sub-domains of CFIR in overall positive studies.

*Denotes the study was counted twice as it had 2 interventions.

**Table 2:**
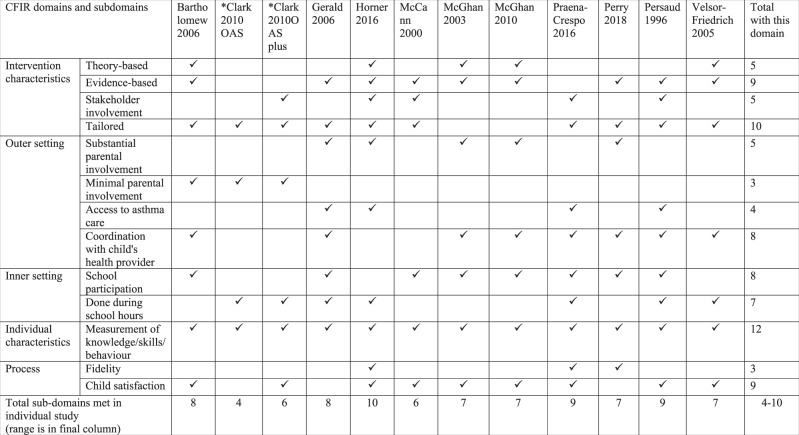
Summary matrix comparing 12 sub-domains of CFIR in no effect studies.

*Denotes the study was counted twice as it had 2 interventions.

All studies with positive effects (12/12) reported parental involvement in their intervention^13–17,21,22,29,30,33–35^ compared to seven studies with no effects (7/11)^18–20,23–25,31^. The Harvest plot (Fig. 2) illustrates the direction of effect with the varying degrees of parental involvement of each study intervention. Studies without parental involvement (including one at low RoB^32^) showed no effect in any of the outcomes of interest^26–28,32^. Of the five interventions with minimal parental involvement^16,18,23,29,34^, the three positive studies were at high RoB and of short duration (≤ 6 months), and either small in sample (study population less than 100 children)^29,34^ or pre/post design^16^. Studies with substantial parental involvement^13–15,17,19–22,24,25,29–31,33,35^ were the only studies to report reduction in absenteeism, though impact on clinical outcomes varied. Cicutto et al.^13^ (cluster RCT at low risk of bias, 170 schools and 1316 children), an example of a study that included parents in care coordination and a showcase at school, had positive effects in school absenteeism and urgent healthcare service use at 12 months. No difference was found in other CFIR subdomains between studies with positive and no effects.

## Discussion

We identified 23 studies (four at low RoB) that evaluated the effectiveness of school-based asthma self-management intervention among primary school children. Twelve of the studies were categorised as being overall positive, though individual outcomes varied; no study reported overall negative impact. The number of CFIR sub-domains addressed varied between studies, but the only component that seemed to be associated with positive outcomes was substantial parental involvement. This was particularly apparent in studies at low RoB.

We found substantial parental involvement to be a crucial component of a school-based asthma self-management intervention among primary school children. Reviews that included interventions targeted at teenagers, in whom parental influence might be expected to be less important, have reached similar conclusions^7,8^. Parental involvement was also found to be important in other school-based interventions for obesity prevention studies^36,37^, self-management of mental health/disorders^38^, and academic enhancement^39^.

However, we did not find other components of interventions (theory-driven, conducted during lesson time, and child satisfaction) to be essential for successful intervention, as was found in the Cochrane review^7^. The differences in the findings were most probably due to a difference in the age group of the children as the Cochrane review included studies among older school children. Our review defined fun, interactive delivery of intervention, as a strategy promoting child satisfaction and engagement, whereas the Cochrane review examined measurement of child satisfaction, an evaluation used mainly in studies targeting adolescents^7^. Primary school children had good participation rates when the sessions were conducted during school hours including during recess, in contrast to adolescents who were less willing to devote their free time including during recess^7,13,16^. Social cognitive and Orem self-care theories were the most used theories, adapted from adults which focuses on self-efficacy and skills of individuals^40–42^. These theories may be suitable for interventions targeting parents and adolescents, but may not be age-appropriate for primary school children with limited decision-making abilities and independent self-management skills^12,43^.

Primary school years are a critical time for children as they spend increasing time away from their parents and begin to learn asthma self-management for themselves^44,45^. Six-year-old children can express opinions, typically reflecting their parents’ actions and views^44^. Over primary school years, they learn from their own experiences and gain the confidence to make independently decisions^44,45^. Although involving parents to support and empower their children’s self-management behaviour is a key concept in the clinical management of children^2,3^, direct parental involvement was not always included in school-based intervention among primary school children^27,28,32^. A key challenge for involving parents is the difficulty of engaging them to attend session(s) delivered in school^24,46^. With the ease of modern telecommunication, alternative methods of engagement such as the use of telephone calls or video sessions could be explored as a convenient alternative to enable substantial parental involvement in the intervention^31,47^.

Although parental involvement is important, an aim of a school-based intervention is to shift the focus of self-management education from parents to children^48,49^. Studies in this review included up to eight educational sessions for children compared to only one to two sessions for parents^13,27,30^. A recent school-based health intervention has recommended the socio-ecological theory where children are the primary focus of an intervention that also involves the children’s social network, e.g. parents, teachers, friends and the school plan/policy^48,50^. Schools could be an ideal setting for this approach, smoothing children’s transition to independent self-management by being located in the child’s environment and including parents as part of the children’s social network^51–53^. Schools also provide a platform for interactive fun groups activities and peer support for children with similar conditions, which could reduce stigma and support self-management practices^13,32^.

The effectiveness of self-management also depends on access and adherence to evidence-based treatments such as controller asthma medications, which is conventionally delivered in healthcare settings^2,5^. ‘Access to healthcare’, however, was a sub-domain least likely to be addressed in the studies included in this review. Although most US-based studies were conducted among minority deprived populations, in whom poor health outcomes may be due to the large disparities in healthcare provision^54^, only five studies reported the access of the children to effective controller medication^15,24,25,27,30^. Even in countries with universal health coverage, such as Canada and United Kingdom, equitable access to high quality healthcare for children cannot be assumed^55^. In low- and middle-income countries, socio-cultural beliefs, physical inaccessibility and lack of education and information are extremely common barriers to healthcare despite universal health coverage^56,57^. Similar barriers are widely described in the US^30,31,34^. Encouragingly, bridging school-based education with the children’s healthcare providers has been a core component of recent school-based interventions^53,58^.

A strength of this review is that we used comprehensive search terms similar to the Cochrane review and searched seven relevant databases. Two reviewers conducted full text screening and data collection was duplicated. A pre-publication update was performed to ensure the findings was up to date this review.

This review has some limitations. Despite a rigorous search strategy, it is possible that we may miss some studies. The screening of title and abstract was conducted by one reviewer, but good agreement resulted after training. Only two studies were conducted in low- and middle-income countries and many studies (15/23) were conducted in the US, reducing generalisability of the review. The included studies were variable in methodologies, instrumentation and data analysis. However, three low RoB studies coincided with the findings and some variability was illustrated in the Harvest plot with the other details described in Supplementary Table 1. Poor reporting of interventions was a challenge and we may have overlooked some intervention components that were not explicitly described. We contacted all the authors to reduce the number of missing information and obtained 39% responses.

A multi-level intervention focusing on the children and involving their social network could provide a useful self-management interventions framework for primary school children and their parents. Specifically, there is a gap in our current understanding of school-based self-management education in younger children in low- and middle-income countries. Future research needs to focus on implementation strategies and effectiveness using this framework. Partnership between schools, parents and healthcare services could create a pragmatic and effective school plan/policy to improve asthma control among children.

School-based self-management interventions for asthma among primary education children can improve asthma outcomes and reduce absenteeism. Parental participation is an important component in this age group, but other features highlighted in secondary school interventions proved less relevant, perhaps reflecting the greater role of parents in younger children.

## Methods

This systematic review follows Cochrane methodology^59^, and PRISMA reporting standards. The protocol is registered with the PROSPERO database (registration number: CRD42019131955).

### Study eligibility criteria

We used a Population, Intervention, Comparator/Control, Outcomes and Study Design (PICOS) strategy to define eligible studies (Table 3)^60^, using definitions similar to the Cochrane review^3,7,61^. Self-management intervention was defined as the active transfer of information to children with asthma to enhance their self-management skills; this was interpreted with reference to components of self-management recommended by global guidelines (Table 3)^2,3^. In line with the Cochrane review, we included non-randomised trials to capture a broader range of studies and thence components used.

**Table 3:** PICO study strategy and definition of terminology.

Participant/population	Children with asthma aged 6–12 years
Intervention	School-based self-management education intervention.
	Definition as active transfer of information to enhance self-management of asthma containing at least one of the core-components of self-management education^2,3^:
	• A basic explanation about asthma, triggers and the factors that influence control
	• Training about correct inhalation technique
	• Information on the importance of the child’s adherence to the prescribed medication regimen
	• Written asthma action plan
	Children with asthma had to be the primary target for the intervention, though others (such as peers without asthma, parents, school staff) could also be included.
Comparator(s)	Standard care or other (non-asthma, or not related to self-management or delayed intervention) education intervention or none
Outcomes	School absenteeism or/and asthma control or/and urgent use of healthcare service
	The definition of the three categories of outcomes of interest were guided by the American Thoracic Society/European Respiratory Society statement^61^:
	1. School absenteeism: Number of days a participant was absent from school (priority due to asthma).
	2. Asthma control: Clinical level of asthma control based on symptoms and capability to perform daily activities measured using asthma symptoms questionnaire/asthma diary with/without objective validation of asthma control, e.g. peak flows or lung function test.
	3. Urgent use of healthcare service: Number of an unscheduled visit to a general practitioner and/or emergency department due to asthma, and the number of days of hospitalisation due to asthma.
Setting	School (primary, elementary or middle school)
Study designs	Experimental study e.g., randomised controlled trial (RCT), cluster RCT, non-randomised study and uncontrolled before-and-after study.

### Outcomes of interest

We chose three outcomes of interest (school absenteeism and two health outcomes - asthma control and urgent use of healthcare services) to reflect the impact on children with poorly controlled asthma^2,7,61^.

### Search strategy

The details of the search terms and databases used are in Supplementary Table 5. The Cochrane review conducted searches in August 2017 using search terms developed by the Cochrane Airway Information Specialist in 23 electronic databases from 1995 onwards and included 55 papers^7^. Using the same search terms, with no language limitations, we updated the search in February 2019 in six-core databases (CENTRAL, MEDLINE, Embase, PsycINFO, CINAHL, AMED)^7^. In addition, we searched the Global Health database using similar search terms without date limits to include studies from low- and middle-income countries. We included all studies identified in the review that met our eligibility criteria (principally excluding those not delivered to primary school children). We checked the reference list and undertook forward citation of studies in the Cochrane review conducted among primary school children^62^.

A pre-publication update was conducted on 17th March 2020 using forward citation of the Cochrane review (published 28 January 2019)^7^ and all the studies included in this review^62^.

### Study selection and data extraction

We imported the list of articles from the electronic databases into Endnote software (version 7) to facilitate screening, de-duplication and overall management of the results. SNR and JS independently screened a random selection of 10% of the titles and abstracts^5^. A 96.3% agreement was achieved prior to discussion, which reached total agreement after clarification of the screening criteria. SNR then completed title and abstract screening. Both reviewers independently conducted full-text screening (which included all the studies in the Cochrane review and those satisfying title and abstract screening), met to discuss discrepancies and decided on the final included papers. Supplementary Table 6 lists studies excluded from this review. A modified Cochrane data extraction form was used for duplicate data extraction (SNR and JS)^63^. SNR contacted authors for missing data by email and any further information received was added to the data extraction forms^59^.

At all stages, any discrepancies not resolved by discussion between the two reviewers were arbitrated by the study team (HP, KEM, LSM, SC).

### Risk of bias of included studies

We used the Cochrane Effective Practice and Organisation of Care (EPOC) Risk of Bias (RoB) tool^64^ to categorise risk into low, high and unclear risk in nine domains, which were then used to generate an overall assessment of the RoB for each study. The Cochrane EPOC RoB tool applies to randomised trials and non-randomised trials^64^. Studies with at least one high-risk domain were summarised as high risk; studies with no high-risk domains but at least one unclear domain were summarised as unclear risk and studies at low risk in all domains were summarised as low risk^64^.

### Data handling

The Consolidated Framework for Implementation Research (CFIR) is a comprehensive framework that systematically identifies factors (sub-domains) that influence the effectiveness of implementation in multi-level interventions^65^. Supplementary Table 3 outlines the 12 CFIR sub-domains. We used CFIR sub-domains to identify context and components in each study (e.g., intervention characteristics, features of the setting and strategies for implementation) that might influence effectiveness of the interventions^66,67^.

We used a structured approach to divide the studies into four categories according to the change in the outcomes of interest^68^. This was a two-step process.

First, we determined the direction of effect in each of the three outcomes of interest (school absenteeism; asthma control; urgent use of healthcare service) for each included study. In some studies, several measures mapped to each outcome of interest: for example, emergency room visits and hospitalisation are both measures of unscheduled care potentially with conflicting findings. The rules at the top of Supplementary Table 1 define how we prioritised outcomes defined as ‘primary’ in the included study, outcomes measured with a validated instrument, and results that were clinically as well as statistically significant. The table then describes how the decision process was applied for each outcome of interest in each study.

Second, we categorised the overall effect of the intervention in each study as positive, negative, no effect or mixed effects, as follows:

- Positive (beneficial): Studies with a positive effect in ≥1 of the outcomes and no negative effects.
- Negative (harmful): Studies with a negative effect in ≥1 of the outcomes and no positive effects.
- No effect: Studies with no positive effects in any of the outcomes.
- Mixed: Studies with at least one positive and one negative outcome.

### Data synthesis

Our preliminary scoping suggested that the studies would be heterogenous in terms of context, components delivered and study design, so we undertook a narrative analysis. We used a Harvest plot^69^ (coded to indicate number of participants, RoB and follow-up duration) to illustrate the effectiveness of the interventions on the three outcomes of interest for each study. A Harvest plot graphically displays not only outcomes but also the weight of the evidence in complex and diverse studies by illustrating selected methodological criteria^69^. We used a matrix to examine the association of the CIFR sub-domains with the overall effectiveness of the interventions. Supplementary Table 4 lists the CFIR sub-domains and how we interpreted them in our analysis.

### Reporting summary

Further information on research design is available in the Nature Research Reporting Summary linked to this article.

## Supplementary information

Supplementary Information

Reporting Summary

## Data Availability

All data that support the findings of this systematic review are already in the public domain.

## Code availability

Authors can confirm that all relevant data codes are included in the paper and/or its supplementary information files.

## References

[1] Global Asthma Network. The Global Asthma Report. (Auckland, New Zealand, 2018).

[2] Global Initiative for Asthma. Global Strategy for Asthma Management and Prevention (updated 2020). (2020).

[3] Scottish Intercollegiate Guidelines Network/British Thoracic Society. SIGN 158 British guideline on the management of asthma. (Scottish Intercollegiate Guidelines Network/British Thoracic Society, London, 2019).

[4] Lougheed MD (2010). Canadian thoracic society asthma management continuum–2010 consensus summary for children six years of age and over, and adults. Can. Respir. J..

[5] Pinnock H (2017). Systematic meta-review of supported self-management for asthma: a healthcare perspective. BMC Med.

[6] Pinnock H (2015). Supported self-management for asthma. Breathe.

[7] Harris, K., et al. School‐based self‐management interventions for asthma in children and adolescents: a mixed methods systematic review. *Cochrane Database of Systematic Reviews* (2019). 10.1002/14651858.CD011651.pub2 PMC6353176 30687940

[8] Walter H (2016). Effectiveness of school-based family asthma educational programs on quality of life and asthma exacerbations in asthmatic children aged five to 18: a systematic review. JBI Database Syst. Rev. Implement. Rep..

[9] Isik E, Fredland NM, Freysteinson WM (2019). School and community-based nurse-led asthma interventions for school-aged children and their parents: a systematic literature review. J. Pediatr. Nurs..

[10] Carvalho Coelho AC, Barretto Cardoso LS, de Souza-Machado C, Souza-Machado A (2016). The impacts of educational asthma interventions in schools: a systematic review of the literature. Can. Respir. J..

[11] Al Aloola NA, Naik-Panvelkar P, Nissen L, Saini B (2014). Asthma interventions in primary schools – a review. J. Asthma.

[12] Orrell-Valente JK, Jarlsberg LG, Hill LG, Cabana MD (2008). At what age do children start taking daily asthma medicines on their own?. J. Pediatr..

[13] Cicutto L, To T, Murphy S (2013). A randomized controlled trial of a public health nurse-delivered asthma program to elementary schools. J. Sch. Health.

[14] Cicutto L (2005). Breaking the access barrier: evaluating an asthma center’s efforts to provide education to children with asthma in schools. Chest.

[15] Levy M, Heffner B, Stewart T, Beeman G (2006). The efficacy of asthma case management in an urban school district in reducing school absences and hospitalizations for asthma. J. Sch. Health.

[16] Magzamen S, Patel B, Davis A, Edelstein J, Tager IB (2008). Kickin’ Asthma: school-based asthma education in an urban community. J. Sch. Health.

[17] Spencer GA, Atav S, Johnston Y, Harrigan JF (2000). Managing childhood asthma: the effectiveness of the open airways for schools program. Fam. Community Health.

[18] Bartholomew LK (2006). Partners in school asthma management: evaluation of a self-management program for children with asthma. J. Sch. Health.

[19] McGhan SL (2003). Evaluation of an education program for elementary school children with asthma. J. Asthma.

[20] McGhan SL (2010). A children’s asthma education program: Roaring Adventures of Puff (RAP), improves quality of life. Can. Respir. J..

[21] Clark NM (2004). Effects of a comprehensive school-based asthma program on symptoms, parent management, grades, and absenteeism. Chest.

[22] Clark NM (2005). A trial of asthma self-management in Beijing schools. Chronic Illn..

[23] Clark NM (2010). An evaluation of asthma interventions for preteen students. J. Sch. health.

[24] Gerald LB (2006). Outcomes for a comprehensive school-based asthma management program. J. Sch. Health.

[25] Horner SD, Brown A, Brown SA, Rew DL (2016). Enhancing asthma self-management in rural school-aged children: a randomized controlled trial. J. Rural Health.

[26] McCann DC, McWhirter J, Coleman H, Calvert M, Warner JO (2006). A controlled trial of a school-based intervention to improve asthma management. Eur. Respiratory J..

[27] Persaud DI (1996). An asthma self-management program for children, including instruction in peak flow monitoring by school nurses. J. Asthma.

[28] Velsor-Friedrich B, Pigott T, Srof B (2005). A practitioner-based asthma intervention program with African American inner-city school children. J. Pediatr. Health Care.

[29] Marsland AL (2019). A randomized pilot trial of a school-based psychoeducational intervention for children with asthma. Clin. Exp. Allergy.

[30] Szefler SJ (2019). Building bridges for asthma care: reducing school absence for inner-city children with health disparities. J. Allergy Clin. Immunol..

[31] Perry TT (2018). Results of an asthma education program delivered via telemedicine in rural schools. Ann. Allergy Asthma Immunol..

[32] Praena-Crespo M, Aquino-Llinares N, Fernandez-Truan JC, Castro-Gomez L, Segovia-Ferrera C (2017). Asthma education taught by physical education teachers at grade schools: a randomised cluster trial. Allergol. et. Immunopathol..

[33] Suwannakeeree P, Deerojanawong J, Prapphal N (2016). School-based educational interventions can significantly improve health outcomes in children with asthma. J. Med. Assoc. Thail.

[34] Isik, E., Fredland, N.M., Young, A. & Schultz, R.J. A school nurse-led asthma intervention for school-age children: a randomized control trial to improve self-management. *J. School Nurs.* **34**, 14–27 (2020). 10.1177/1059840520902511 32148181

[35] Simoneau T (2020). A school nurse-led asthma program reduces absences: evaluation of easy breathing for schools. Acad. Pediatr..

[36] Verjans-Janssen SRB, van de Kolk I, Van Kann DHH, Kremers SPJ, Gerards S (2018). Effectiveness of school-based physical activity and nutrition interventions with direct parental involvement on children’s BMI and energy balance-related behaviors - a systematic review. PLoS ONE.

[37] Golley RK, Hendrie GA, Slater A, Corsini N (2011). Interventions that involve parents to improve children’s weight-related nutrition intake and activity patterns – what nutrition and activity targets and behaviour change techniques are associated with intervention effectiveness?. Obes. Rev..

[38] Shucksmith J, Jones S, Summerbell C (2010). The role of parental involvement in school-based mental health interventions at primary (Elementary) school level. Adv. Sch. Ment. Health Promotion.

[39] Lara L, Saracostti M (2019). Effect of parental involvement on children’s academic achievement in Chile. Front Psychol..

[40] Bandura, A. Social Cognitive Theory. In *Annals of child development*, Vol. 6 (1989).

[41] Ng CY (2018). Theory-based health behavior interventions for pediatric chronic disease management: a systematic review. JAMA Pediatr..

[42] Orem, D. *Nursing: Concepts of practice*, (Mosby, 2001).

[43] Horner SD (1998). Using the Open Airways curriculum to improve self-care for third grade children with asthma. J. Sch. Health.

[44] Buford TA (2004). Transfer of asthma management responsibility from parents to their school-age children. J. Pediatr. Nurs..

[45] Ramdzan SN (2020). How young children learn independent asthma self-management: a qualitative study in Malaysia. Arch. Dis. Child..

[46] Okely AD, Hammersley ML (2018). School-home partnerships: the missing piece in obesity prevention?. Lancet Child Adolesc. Health.

[47] Frey SM, Milne Wenderlich A, Halterman JS (2019). New opportunities with school-based telehealth: convenient connections to care. JAMA Pediatrics.

[48] Nuss HJ (2016). Applying the social ecological model to creating asthma-friendly schools in Louisiana. J. Sch. Health.

[49] Kirk S (2013). The effectiveness of self-care support interventions for children and young people with long-term conditions: a systematic review. Child. Care Health Dev..

[50] Van Koperen TM (2013). Characterizing the EPODE logic model: unravelling the past and informing the future. Obes. Rev..

[51] Mukamana O, Johri M (2016). What is known about school-based interventions for health promotion and their impact in developing countries? A scoping review of the literature. Health Educ. Res..

[52] Szefler SJ (2020). A worldwide charter for all children with asthma. Pediatr. Pulmonol..

[53] Cicutto, L., et al. Building bridges for asthma care program: a school-centered program connecting schools, families, and community health-care providers. *J. School Nurs.* **36**, 168–180 (2018). 10.1177/1059840518805824 PMC7222283 30336726

[54] Holsey CN, Collins P, Zahran H (2013). Disparities in asthma care, management, and education among children with asthma. Clin. Pulm. Med..

[55] Cylus J, Papanicolas I (2015). An analysis of perceived access to health care in Europe: how universal is universal coverage?. Health Policy.

[56] Sanogo NA, Fantaye AW, Yaya S (2019). Universal health coverage and facilitation of equitable access to care in Africa. Front. Public Health.

[57] Kan XH (2012). Asthma as a hidden disease in rural China: opportunities and challenges of standard case management. Public Health Action.

[58] Frey SM, Halterman JS (2017). Improving asthma care by building bridges across inpatient, outpatient, and community settings. JAMA Pediatrics.

[59] Higgins, J. P. T. & Green, S. (editors). Cochrane Handbook for Systematic Reviews of Interventions Version 5.1.0 [updated March 2011], (The Cochrane Collaboration, 2011).

[60] Santos CMC, Pimenta CAM, Nobre MRC (2007). The PICO strategy for the research question construction and evidence search. Rev. Lat..

[61] Reddel HK (2009). An official American Thoracic Society/European Respiratory Society statement: asthma control and exacerbations: standardizing endpoints for clinical asthma trials and clinical practice. Am. J. Respir. Crit. Care Med..

[62] Greenhalgh T, Peacock R (2005). Effectiveness and efficiency of search methods in systematic reviews of complex evidence: audit of primary sources. BMJ.

[63] Cochrane Effective Practice and Organisation of Care (EPOC). EPOC Resources for Review authors. in Screening, data extraction and management (2017).

[64] Cochrane Effective Practice and Organisation of Care (EPOC). Suggested risk of bias criteria for EPOC reviews. EPOC Resources for review authors. (2017).

[65] Damschroder LJ (2009). Fostering implementation of health services research findings into practice: a consolidated framework for advancing implementation science. Implement. Sci..

[66] Snilstveit B, Oliver S, Vojtkova M (2012). Narrative approaches to systematic review and synthesis of evidence for international development policy and practice. J. Dev. Effectiveness.

[67] Keith RE, Crosson JC, O’Malley AS, Cromp D, Taylor EF (2017). Using the Consolidated Framework for Implementation Research (CFIR) to produce actionable findings: a rapid-cycle evaluation approach to improving implementation. Implement Sci..

[68] Burns J (2018). Looking beyond the forest: using harvest plots, gap analysis, and expert consultations to assess effectiveness, engage stakeholders, and inform policy. Res. Synth. Methods.

[69] Ogilvie D (2008). The harvest plot: a method for synthesising evidence about the differential effects of interventions. BMC Med. Res. Methodol..

