# Publisher Correction: School-based self-management interventions for asthma among primary school children: a systematic review

**DOI:** 10.1038/s41533-021-00240-0

**Published:** 2021-05-06

**Authors:** Siti Nurkamilla Ramdzan, Julia Suhaimi, Katherine M. Harris, Ee Ming Khoo, Su May Liew, Steve Cunningham, Hilary Pinnock

**Affiliations:** 1 Department of Primary Care Medicine, Faculty of Medicine, University of Malaya, Kuala Lumpur, Malaysia; 2 NIHR Global Health Research Unit on Respiratory Health (RESPIRE), Usher Institute, University of Edinburgh, Edinburgh, UK; 3 Centre for Child Health, Blizard Institute, Queen Mary University of London, London, UK

**Keywords:** Asthma, Paediatrics

Correction to: *npj Primary Care Respiratory Medicine* 10.1038/s41533-021-00230-2, published online 1 April 2021

The original version of the published Article contained an error in the data of Tables 1 and 2.

This has been corrected in the HTML and PDF version of the Article.

