# Supplementary material for: School-based self-management interventions for asthma among primary school children: a systematic review
Source: NPJ Prim Care Respir Med. 2021 Apr 1;31:18. doi: 10.1038/s41533-021-00230-2 (PMC8016947; doi:10.1038/s41533-021-00230-2)
Supplement: Supplementary file 1 — Supplementary Information [file 41533_2021_230_MOESM1_ESM.pdf]

Supplementary Table 1: Summary of study, CFIR domains and researchers' interpretation of included studies

Where outcomes within a category were conflicting, the decision process attached priority as follows:

- Defined primary outcomes in an adequately powered study
- Outcomes which were measured with a validated instrument (as opposed to responses of non-validated instrument)
- Outcomes that were clinically as well as statistically significant (e.g. defined as minimum clinically important difference)
- Outcomes which were measured using continuous versus categorical/dichotomy scale (e.g. days of school absenteeism versus yes/no to school absenteeism in a year)
- Outcomes reported by children as opposed to parents (in the absence of a validated instrument which measured both)

Finally, if there were any remaining doubts, the authors' interpretation was considered as providing the context for our decision

Citation design, size, risk of bias, and intervention	CIFR domains (ˣ indicates component of study not fulfilling criteria)	Reported outcomes (* indicates primary outcome if stated)	Researchers' interpretation for Harvest plot
STUDIES WITH OVERALL POSITIVE EFFECT			
Cicutto, 2005[14]<br>cRCT FU:12m<br>Canada. Urban<br>26 schools: 256 children, Age: 6-11yrs<br><br>Intervention: Roaring Adventures of Puff<br><br>RoB: Low	Intervention characteristics<br>• Social cognitive theory and self-regulation<br>• Based on Canadian guideline<br>• Involved HCPs and families in development<br>• Tailored at individual level<br>Outer setting<br>• Parents attended showcase + homework<br>• Universal Health Coverage<br>• Communicated/coordinated with HCP<br>Inner setting<br>• ˣ Only permission/advertisement at schools<br>• Delivered mostly during lunch/class time<br>Individual characteristics<br>• Self-efficacy assessed<br>Process<br>• Fidelity: Implemented as designed<br>• Puppetry, games, role-play, model building, etc	School absenteeism<br>Days lost from school: Significant between group difference<br>• Mean (SD) days/yr: I: 3.0 ±4.4 vs C: 4.3 ±5.7 (p<0.05)	Significant reduction in missed school in intervention group<br><br>Illustrated as positive effect
		Asthma control<br>Not reported	
		* Urgent use of healthcare services<br>Number of urgent health care visits (ED, walk-in or same-day visits)<br>Significant between group difference<br>• Mean (SD) visits/yr: I: 1.7 (1.9) vs C: 2.5 (2.5), p <0.01	Significant reduction in urgent healthcare visits in intervention group<br>Illustrated as positive effect
Cicutto, 2013[13]<br>cRCT FU:12m<br>Canada.	Intervention characteristics<br>• Social cognitive theory<br>• Based on Canadian guideline<br>• Developed by schools	School absenteeism<br>Proportion of children with asthma-related absence.<br>Significant between-group reduction in proportion with<br>• any school absence: I: 50% vs C: 60% (p=0.01)	Significant reduction in absenteeism in intervention group<br>Illustrated as consistently positive effect.

170 schools; 1316 children, Age 6-11yrs<br><br>Intervention: Roaring Adventures of Puff + asthma resource kit<br><br>**RoB: Low**	• Locally tailored<br>Outer setting<br>• Parents attended showcase + coordination of care<br>• Universal health coverage<br>• Letter to HCP via family<br>Inner setting<br>• Resource kit and school community session<br>• Delivered during lunch time<br>Individual characteristics<br>• Inhaler technique assessed<br>Process<br>• Fidelity: Implemented as designed<br>• Interactive games, puppetry, art, skits, homework	• >20 missed school days: I: 1.4% vs C: 4.5% p-value= 0.01	
		Asthma control<br>Not reported	
		* Urgent use of healthcare services<br>Proportion of children attending urgent care. Significant between-group reduction in:<br>• * Any urgent care: I: 41.3% vs C: 51.4% (p=0.0001)<br>• Unscheduled physician's visit: I: 24.1% vs 31.2% (p=0.001)<br>• ED attendances: I: 8.2% vs 2.8% (p=0.02)<br>Walk-in clinic use: I: 18.4% vs 21.6%, p=NS	Significant reduction in use of healthcare resources in intervention group.<br><br>Illustrated as positive effect
Clark, 2005[22]<br>cRCT. FU:12m<br>China. Urban/rural.<br>21 schools: 639 children, Age 7-11<br><br>Intervention: Tailored Open Airway for School<br><br>**RoB: Unclear**	Intervention characteristics<br>• Social cognitive theory<br>• Based on GINA & NAEPP<br>• Adapted to local needs<br>Outer setting<br>• School fair for parents with Q& A session<br>• [x] HCP requested to provide PAAP<br>Inner setting<br>• Session for school principals and counsellors<br>• Individual characteristics<br>• Parent management index of items<br>Process<br>• [x] Fidelity: good except for HCP response<br>• Games, problem solving exercises	School absenteeism<br>Days lost from school: Significant between group reduction in:<br>• adjMean diff days/yr I: -0.32 vs C: -0.56 (p=0.02)	Significantly fewer days lost from school in the intervention group.<br>Illustrated as positive effect
		Asthma control<br>Days with symptoms: No significant between group difference<br>• **adjMean diff days/yr I: -9 vs C: -6 p=0.13**	No significant effect of the intervention on days with symptoms.<br>Illustrated as no effect
		Urgent use of healthcare services<br>Number of hospitalisations or ED visits: no significant between group difference in odds of a reduction in:<br>• Hospitalisations: adjOR 1.43 (p=0.36)<br>• ED attendances: adjOR 1.00 (p=0.98)	No significant effect of intervention on hospitalisations or ED visits.<br>Illustrated as no effect
Clark, 2004[21]<br>cRCT, FU: 24m<br>USA: Urban, minority<br>14 schools: 835 children, Age: 7-11yrs<br><br>Intervention: Tailored Open Airway for School plus	Intervention characteristics<br>• Tailored to local needs<br>Outer setting<br>• School fair and assignments to include parents<br>• [x] contact HCP (not successfully done)<br>Inner setting<br>• Session with principle and counsellors<br>Individual characteristics	School absenteeism<br>Proportion of children with asthma-related absence. Significant between-group reduction in:<br>• **School absence:** I: reported 8% fewer absences than C (p<0.05)	Significant reduction in asthma related absence in intervention group.<br>Illustrated as positive effect
		Asthma control<br>Proportion reporting symptoms. Significant between-group difference in the relative change of adjusted:<br>• Day symptoms: I reported 17% fewer symptoms than C (p=0.0001)<br>(Persistent symptoms; 14% fewer; Intermittent symptoms 22% fewer)	Unclear definitions of 'symptomatic', contradicting results, and limited reporting of data (no absolute values)

RoB: High	• Parent management index<br>Process<br>• [X] one key element not successful (contact HCP)	• Night symptoms: I reported 40% more symptoms than C (p<0.0001) (Persistent symptoms; 14% fewer; Intermittent symptoms 255% more)	Illustrated as no effect but hatched to indicate inconsistency
		Urgent use of healthcare services<br>Not reported	
Isik, 2020[34]<br>RCT FU: 12 weeks<br>USA, Urban minority<br>8 schools: 73 children<br>Age: 7-12yrs<br>Intervention: School Nurse-led<br><br>RoB: High	Intervention characteristics<br>• Orem self-care theory<br>• Based on ALA guideline<br>• Developed with school nurse<br>• Tailored to children's condition<br>Outer setting<br>• Parental received information sheet<br>• [X] assessment of asthma care access<br>• [X] Coordination with HCP<br>Inner setting<br>• [X] school staff participation<br>• Delivered during school hours<br>Individual characteristics<br>• Asthma management plan<br>Process<br>• Fidelity: implemented as planned<br>• Storytelling, colouring, drawing, etc	School absenteeism<br>Number of absences. No significant between group difference in:<br>• mean (SD) all cause absences I: 1.3 (1.6) vs C:1.8 (1.5), p=0.179	No significant effect of intervention on all cause of school absenteeism<br>Illustrated as no effect.
		Asthma control<br>Validated symptom score and control. Significant difference between group difference at baseline to 6 week and baseline to 12 weeks in:<br>• Mean ACQ scores: F (2, 138) = 14.2, p< 0.001	No data provided: Authors stated significant improvement in symptoms in intervention group<br>Illustrated as positive effect
		Urgent healthcare services<br>Not reported	
Levy, 2006[15]<br>cRCT, step wise<br>FU: 12m<br>USA, Urban minority<br>20 schools: 329 children, Age: 6-10yrs<br><br>Intervention: Open Airway for School plus<br><br>RoB: High	Intervention characteristics<br>• Based on US guideline<br>Outer setting<br>• Parents in coordination care + follow-up calls<br>• 80% of children were insured<br>• Contacted HCP<br>Inner setting<br>• Training for school staff + dialogues if needed<br>Individual characteristics<br>• Asthma knowledge<br>Process (none)	*School absenteeism<br>Days lost from school: Significant between group difference in:<br>• Mean days/school yr: 4.38 vs C:8.18	No p value provided: Authors stated 'significant improvement in school attendance'.<br>Illustrated as positive effect
		Asthma control<br>Not reported	
		*Urgent healthcare services<br>Significant between-group difference mean number of visits in<br>• Mean (SD) Urgent/ED visits. I:1.36 (0.49) vs C: 1.59 (1.0), p<0.0001<br>• Mean days in hospital, I: 0.18 (0.73) vs C: 0.45 (1.06), p <0.05	Significant fewer in urgent care, ED visits and hospitalisations in intervention group<br>Illustrated as consistently positive effect

Magzamen, 2008[16]<br>Uncontrolled study<br>FU: 3 m<br>USA, Urban minority<br>18 schools: 990 children (3-yr groups), Age:11-12 yrs<br><br>Intervention: Kickin' Asthma [Delivered to three year groups (YG1/2/3)<br><br>RoB: High	Intervention characteristics<br>• School staff and children involved in development<br>• Developed by local schools<br>Outer setting<br>• Customised letter to parents<br>Inner setting<br>• [X] School staff delivered/received intervention<br>• Delivered during lunchtime<br>Individual characteristics<br>• Asthma management behaviour + spacer technique<br>Process<br>• Skits, games, videos, role-play	School absenteeism<br>Days lost from school: Variable within-group impact in the three-year groups (YG) in:<br>• Days /3m mean diff (SE): YG1 (reduced): -0.54 (0.30), p<0.03; YG2 (no effect): -0.26 (0.12), p<0.1; YG3 (no effect): -0.08 (0.13) p=0.44	Significant reduction in school absenteeism in one of the three-year groups.<br>Illustrated as no effect but hatched to indicate inconsistency
		Asthma control<br>Activity limitation past 4 weeks. Significant within-group reduction in all year groups (YGs)<br>• Days/4w mean diff (SE) YG1: -0.70 (0.36), p<0.006; YG2: -0.62 (0.34), p<0.0001; YG3: -1.12 (0.37) p<0.0001<br>Night-time symptoms past 4 weeks. Significant within-group reduction in all year groups (YGs)<br>• Nights/4w mean diff (SE) YG1: -0.99 (0.29), p<0.006; YG2: -0.68 (0.29), p<0.0001; YG3: -0.43 (0.40) p=0.005	Significant reduction in asthma symptoms in all year groups after intervention<br><br>Illustrated as consistently positive effect
		Urgent healthcare services<br>Episodes of urgent care. Significant within-group reduction in all year groups (YGs) in odds of:<br>• One or more ED visit or hospitalisation: OR (95% CI): YG1: 3.13 (1.41-6.92), YG2: 3.83 (2.03-7.23), YG3: 2.36 (1.26-4.40)<br>• One or more unscheduled GP visit OR (95% CI): YG1: 3 .00 (1.41-6.39), YG2: 2.5 (1.59-3.93), YG3: 1.21(0.74-2.00)	Significant reduction in ED, hospitalisations and unscheduled GP visits in all year groups after intervention<br><br>Illustrated as consistently positive effect
Marsland, 2019[29]<br>RCT, FU: 4 m<br>USA, Urban minority<br>12 schools: 104 children<br>Age: 8-14 (mean: 10.6)<br><br>Intervention: I Can Cope (ICC) and Open Airway for Schools (OAS)<br><br>RoB: High	Intervention characteristics<br>• ICC: Life-stress theory<br>• [X] based on evidence-based guideline<br>• ICC: Developed with HCP and school nurse<br>• [X] tailored to culture/beliefs<br>Outer setting<br>• ICC: Parental attended session + received letter/call<br>• OAS: Based on US guideline<br>• [X] assessment of asthma care<br>• [X] coordination with HCP<br>Inner setting<br>• ICC: Teachers involved in development and coordination<br>• Delivered during school hours	School absenteeism<br>Not measured	
		Asthma control<br>Validated asthma control rated by child. Significant between-group difference in mean score post-intervention:<br>CHSDA-C Mean (SD): ICC:1.2 (2.0) vs C:2.6 (2.3), p<0.05; OAS: 1.4 (2.1) vs C: 2.6 (2.3), p<0.05<br>Asthma control rated by parents. No significant between-group difference in mean score post-intervention<br>• CHSA Mean (SD) I: 1.2 (1.3) vs C: 1.7 (2.2) p=NS; OAS: 1.4 (2.1) vs 1.7 (2.2)	Children reported asthma prioritised due to the age of the children. Significant reduction in asthma control in intervention groups<br><br>Illustrated as positive but inconsistent (Hatched)

	Individual characteristics<br>• Management self-management score<br>Process<br>• Fidelity: implemented as planned<br>• ICC: Games and interactive activities, OAS: Games and stories	Urgent healthcare services<br>Not reported	
Simoneau, 2020[35]<br>Non-randomised study<br>FU: 12 m<br>USA, Urban minority<br>15 schools; 251 children<br>Intervention: Easy Breathing for Schools<br><br>RoB: High	Intervention characteristics<br>• Based on opinion from nurse, parents and HCPs<br>Outer setting<br>• Parent attended session at school<br>• Communication with child HCP<br>Inner setting<br>• School nurse delivered intervention<br>Individual characteristics<br>• Inhaler technique delivered and assessed<br>Process<br>• [x] Fidelity: only 25% implemented 3 core elements	*School absenteeism<br>Days lost from school: Significantly lower between-group risk of:<br>• Days absent: adjRR=0.75 (95%CI 0.67 to 0.85) p<0.001	Adjusted for sex, ethnicity, age, and school year. 25% fewer absences in intervention group.<br>Illustrated as positive effect
		Asthma control<br>Not reported	
		Urgent healthcare services<br>Not reported	
Spencer, 2000[17]<br>Uncontrolled study<br>FU: 6 m<br>USA.<br>40 schools: 369 children, Age: 6-13 yrs<br><br>Intervention: Open Airway for School<br><br>RoB: High	Intervention characteristics<br>• (None)<br>Outer setting<br>• Parent attended session at school<br>Inner setting<br>• (None)<br>Individual characteristics<br>• Management of asthma symptoms<br>Process<br>• (None)	School absenteeism<br>Parent-reported absences. No significant within-group difference<br>• % with ≥1 absence/6m: Pre: 53% vs post: 53%, p=NS<br>School days missed (nurse-reported) Significant within-group reduction:<br>• Mean days/6m. Pre: 5.50 vs post: 3.73. (p<0.001)	Significant effect of intervention on missed school days, but not proportion with an absence.<br>Illustrated as positive effect but hatched to illustrate inconsistency
		*Asthma control<br>Asthma symptoms. Significant within group difference improvement<br>• Mean score: Pre: 25.9 vs post: 23.9 (p<0.001)	Significant reduction in non-validated symptom score<br>Illustrated as positive effect
		Urgent healthcare services<br>Parent-reported events Significant within-group difference in:<br>• % with ≥1 ED visit/6m: Pre: 33% vs post: 18%, p<0.001<br>• % with ≥1 hospitalisation: Pre: 14% vs post: 7%, p=0.002<br>Event (nurse-reported) Significant within-group difference in:<br>• Number of ED visits (Mean): Pre: 0.71 vs post: 0.18, p<0.001<br>• Number of hospitalisations (Mean): Pre: 0.14 vs post:0.04, p<0.013	Significant reduction in ED visits and hospitalisation after intervention<br>Illustrated as consistently positive effect
Suwannakeeree, 2016[33]<br>Uncontrolled study	Intervention characteristics<br>• Based on US guideline<br>• Individually tailored to each child	School absenteeism<br>School absences. Significant within-group difference<br>• % (n) with ≥1 absence/6m: Pre: 48% (14) vs post: 17% (5) p=0.004	Significant reduction in missed school after intervention<br>Illustrated as positive effect

FU:6 m<br>Thailand, Urban<br>1 school: 29 children<br>Age: 6-12 (extracted sub-analysis)<br><br>Intervention: Asthma Friendly School Initiative<br><br>RoB: High	Outer setting<br>• Parents attended sessions at school<br>• Medication provided to all participants<br>• Sent spirometry results + other information to HCP<br>Inner setting<br>• Teachers were trained asthma management plan<br>• Delivered during school hours<br>Individual characteristics<br>• Asthma management behaviour<br>Process<br>• [X] fun interactive activity	*Asthma control<br>Symptoms. Significant within-group increase in proportion with:<br>• Day symptoms ≤2/wk %(n): Pre: 48% (14) vs post: 90% (26), p<0.001<br>• No night symptom %(n): Pre: 59% (17) vs post: 83% (24), p = 0.020<br>SABA use: No significant within-group difference in proportion using<br>• SABA ≤2/wk % (n): Pre: 100% (29) vs post: 90% (26) p=0.25	Significant reduction in asthma symptoms but no significant effect on bronchodilator use after intervention<br>Illustrated as positive effect but hatched to show inconsistency
		*Urgent healthcare services<br>ED visits. Significant within-group reduction n proportion with:<br>• ≥1 ED visit % (n): Pre: 59% (17) vs post: 21% (6), p=0.002	Significant reduction in ED visit after intervention<br>Illustrated as positive effect
Szefler, 2019[30]<br>Non–randomised study, FU: 12m<br>USA, minority<br>463 children, Age: 5-14 (89.7% between 6-12)<br><br>Intervention: Building Bridges<br><br>RoB: High	Intervention characteristics<br>• Based on NAEPP guideline<br>• School staff and nurse involved in development<br>• Tailored to culture/beliefs<br>Outer setting<br>• Parental attended session<br>• 85% participant had medical insurance<br>• Letter to HCP<br>Inner setting<br>• School nurses delivered intervention<br>• Delivered during school hours<br>Individual characteristics<br>• Inhaler technique score<br>Process<br>• Fidelity: implemented as planned<br>• Interactive session	School absenteeism*<br>School days missed. Significant between-group reduction in absenteeism<br>• Mean % school days missed: I: 9% vs C: 12% p<0.001.	Significant reduction in absenteeism in intervention group<br>Illustrated as positive effect
		Asthma control<br>Validated control test: Significant within-group increase in ACT:<br>• ACT Mean (SD): Pre: 19.5 (0.2) vs post: 21.1 (0.2), p<0.01<br>Proportion poorly controlled: Significant within-group reduction in proportion of children below the ACT threshold for good control<br>• % with ACT score ≤19: Pre: 43% vs post: 29%, p <0.01	Significant reduction in asthma control after intervention<br><br>Illustrated as positive effect
		Urgent healthcare services<br>Not reported	

Citation design, size, risk of bias, and intervention	CIFR domains	Reported outcomes (* indicate primary outcome if stated)	Researchers' interpretation for Harvest plot
STUDIES WITH OVERALL NO EFFECT			
McGhan, 2010[20]<br>cRCT FU: 12m<br>Canada, Majority<br>34 schools: 266 children, Age: 6-13 (8.6)<br><br>Intervention: Roaring Adventures of Puff<br><br>RoB: Low	Intervention characteristics<br>• Social cognitive theory<br>• Based on Canadian guideline<br>Outer setting<br>• Parents attended session at school<br>• Letter + communication with HCP<br>Inner setting<br>• Session for teachers<br>Individual characteristics<br>• Medication use and management behaviour<br>Process<br>• Puppetry, games, videos, role-play, etc	School absenteeism<br>School days missed. No significant between-group difference in<br>• Missed school days/yr (Mean): I: 4.0 vs C: 2.5 (NS)	No significant effect of intervention on missed school days<br>Illustrated as no effect
		Asthma control<br>Not reported	
		Urgent healthcare services<br>No significant between group difference in mean of:<br>• Unscheduled visit (Mean): I: 1.2 vs C: 0.7 (NS)<br>• ED visits (Mean): I: 0.2 vs C: 0.07 (NS)	No significant effect of intervention on unscheduled visits and ED visits.<br>Illustrated as consistently no effect
Praena-Crespo, 2017[32]<br>cRCT FU: 6 months<br>Spain<br>97 schools:381 children<br>Age: 10-12yrs<br>Intervention: Asthma, Sport and Health programme<br><br>RoB: Low	Intervention characteristics<br>• [X] Evidence-based (used expert consensus)<br>• Developed jointly with teachers and HCP<br>• Tailored to Spanish children<br>Outer setting<br>• [X] Parental involvement<br>• Universal Health Coverage<br>• Coordination with HCPs using forms<br>Inner setting<br>• PE teachers delivered intervention<br>• Delivered during PE period<br>Individual characteristics<br>• Newcastle asthma knowledge questionnaire<br>Process<br>• Implemented as planned<br>• Video and slides presentation	School absenteeism<br>School attendance from Education authority.<br>"The intervention programme decreases absenteeism, without reaching significance"	No data provided: authors stated no significant between group difference<br>Illustrated as no effect
		Asthma control<br>Validated asthma control questionnaire No significant between-group difference in:<br>• CAN score Mean (95%CI): I: 11.25 (9.93 to 12.57) vs C: 10.61 (9.43 to 11.78), p =NS	No significant difference between control and intervention.<br><br>Illustrated as no effect
		Urgent healthcare services<br>Not reported	

Bartholomew, 2006[18]<br>cRCT FU: 12m<br>USA, Urban minority,<br>60 schools: 946 children, Age: 6-10yrs (Grade 1-4)<br><br>Intervention: Partners in School Asthma Management program<br><br>RoB: Unclear	Intervention characteristics<br>• Social cognitive theory<br>• Based on NAEPP<br>• Tailored to language, health literacy<br>Outer setting<br>• Parents mailed action plans, video<br>• Tailored letters and video to HCP<br>Inner setting<br>• School action committee<br>Individual characteristics<br>• Self-efficacy assessed<br>Process<br>• Interactive computer program	School absenteeism<br>Missed school in days' Overall rates of absenteeism declined but here were no between-group differences in the frequency of absences over time'	No between-group data provided: Authors state no significant between-group difference<br>Illustrated as no effect
		Asthma control<br>Validated Usherwood Symptom Questionnaire. 'Symptoms declined significantly over time, but there were no between-group differences on symptom level or rate of decline'	No between-group data provided: Authors state no significant between-group difference<br>Illustrated as no effect
		Urgent use of healthcare services<br>Hospitalisations/ED. "Increasing over time but there was no between-group difference in the level or rate of increase of hospitalisations at post-test by group."	No data provided: Authors state no significant between-group difference<br>Illustrated as no effect
Clark, 2010[23]<br>cRCT, FU: 12m<br>USA. Urban, minority<br>19 schools: 1292 children, Age: 10-13 (mean: 11.6)<br>Intervention: tailored Open Airway for School (OAS) and tailored OAS + peer component (OAS+)<br>RoB: Unclear	Intervention characteristics<br>• OAS+: Children involvement<br>• Adapted to minority in urban setting<br>Outer setting<br>• Take home assignments and material for parents<br>Inner setting<br>• [x] School staff delivered/received intervention<br>• Delivered during school hours<br>Individual characteristics<br>• Asthma self-regulation and management scale<br>Process<br>• OAS+: Games, role-play, artistic activities	School absenteeism<br>Not reported	
		*Asthma control<br>Symptoms score No significant differences between either group and control in mean frequency of day symptoms.<br>• Odds of a fall in day-time symptoms OAS: OR = 1.1 (p> 0.5) OAS+: OR = 1.3 (p=0.3). No data provided for control group	No significant difference between intervention A and B with control groups for symptoms.<br>Illustrated as no effect
		Urgent healthcare services<br>Not reported	
Gerald, 2006[24]<br>cRCT FU:12m<br>USA, Urban minority<br>54 schools: 736 children, Age: 6-10 yrs<br>Intervention: tailored Open Airway for School + educational programme for school community<br><br>RoB: Unclear	Intervention characteristics<br>• Included written personalised asthma action plan<br>• Tailored asthma plan individually to each child<br>Outer setting<br>• Parent attended session at school<br>• All children received asthma medication<br>• Coordination care with school nurse and HCP<br>Inner setting<br>• Session for school faculty and staff<br>• Delivered during physical education period<br>Individual characteristics	*School absenteeism<br>Number of all-cause absences. No significant between-group difference<br>Number of absences Mean (SD) I: 3.88 (3.5) vs C: 3.21 (3.2), p=NS	No significant effect of intervention on school absenteeism<br>Illustrated as no effect
		Asthma control<br>Not reported	
		Urgent healthcare services<br>Number of ED and hospital events. No significant between group difference:<br>• ER visits Median (SD): I: 0.09 (0.28) vs C: 0.10 (0.31), NS<br>• Hospitalisations (median (SD): I: 0.04 (0.19) vs C: 0.02 (0.14), NS	No significant effect of intervention on ER visit and hospitalization.<br>Illustrated as consistently no effect

	• Knowledge score<br>Process<br>• [X]Fidelity: program shortened, not as planned		
Horner, 2016[25]<br>cRCT, FU:12m<br>USA, Rural minority<br>33 schools: 168 children, Age: 7-11 yrs<br>Intervention: Asthma Plan for Kids [Only the school-based group extracted for this review]<br><br>RoB: unclear	Intervention characteristics<br>• Bruhn's theoretical Model<br>• Based on US guideline<br>• Parents involved in development<br>• Tailored to families in rural areas<br>Outer setting<br>• Group presentation and booklets for parents<br>• 92% of children were insured<br>• [X] Coordination care with HCP<br>Inner setting<br>• [X] school involvement<br>• Delivered during lunch time<br>Individual characteristics<br>• Asthma management score<br>Process<br>• Fidelity: measured<br>• Vignettes + problem solving	School absenteeism<br>Not reported	
		Asthma control (School-based intervention only)<br>Asthma symptoms No significant-between group difference:<br>• Severity of chronic asthma scale Mean (SD) : I: 3.38 (0.69) vs C: 3.79 (1.34), NS	No significant effect of intervention on asthma control<br>Illustrated as no effect
		*Urgent healthcare services (School-based intervention only)<br>Urgent asthma events. No significant between-group difference in:<br>• *Office visits/12m Mean (SD) I: 0.49 (1.02) vs C: 0.69 (1.3), NS<br>• *Hospitalisation/12m Mean (SD) I: 0.01 (0.11), vs C: 0.02 (0.22), NS<br>• *ED visits/12m Mean (SD): I: 0.04 (0.19) vs C: 0.04 (SD 0.19), NS	No significant effect of intervention on offices visits, hospitalisation and ED visits<br>Illustrated as no effect
McCann, 2006[26]<br>cRCT FU : 12 m<br>UK<br>24 schools: 219 children, Age: 7-9 yrs<br>Intervention: Nurse-led<br><br>RoB: Unclear	Intervention characteristics<br>• Based on UK guideline<br>• Developed with school staff<br>• Tailored to local evidence<br>Outer setting [None]<br>Inner setting<br>• Session for teachers<br>Individual characteristics<br>• Asthma knowledge + self-confidence + self-esteem<br>Process<br>• Role play	*School absenteeism<br>School days missed. 'After adjusting for social deprivation, no effect of the intervention was found.'<br>• Within intervention group: Mean days/yr (SD) Pre: 7.0 (7.4) vs post 6.8 (6.1) [No control group data provided]	No data provided: authors state no significant effect of intervention on school absenteeism<br>Illustrated as no effect
		Asthma control<br>Asthma symptoms reporting. 'Resolution of symptoms for all children (Chi-squared 21.8; p=0.0005), but no effect of the intervention was found.'	No data provided: authors state no significant effect of intervention on asthma control<br>Illustrated as no effect
		Urgent healthcare services<br>Not reported	
McGhan, 2003[19]<br>cRCT FU:<br>Canada, Majority<br>18 schools: 162 children, Age: 5-13	Intervention characteristics<br>• Social cognitive theory<br>• Based on Canadian guideline<br>Outer setting<br>• Parents attended session at school	School absenteeism<br>School absences. No significant between-group difference in:<br>% (n) with $\geq$1 absence/12m: I: 39% vs C: 47% (p=0.07)	No significant effect of intervention on missed school days<br>Illustrated as no effect
		Asthma control	No significant difference of intervention for all measurement

Intervention: Roaring Adventures of Puff<br><br>RoB: Unclear	• Letter + communication with HCP<br>Inner setting<br>• Session for teachers<br>Individual characteristics<br>• Self-efficacy and management behaviour<br>Process<br>• Puppetry, games, role-play, model building etc	Reported symptoms. Mixed findings: Only one significant between-group difference in proportion of children in last 2w with:<br>• Waking with symptoms: I: 45 % vs C: 39% (p>0.1)<br>• Coughing (mod/severe: I: 25% vs C: 28% (p>0.1)<br>• Tight chest (mod/severe): I: 11% vs C: 11% (p>0.1)<br>• Wheezing (mod/severe): I: 15% vs C: 14% (p>0.1)<br>• Shortness of breath (mod/severe): I: 15% vs C: 11% (p>0.1)<br>• Limited kind of play: I: 29% vs C: 31% (p<0.01)<br>• Limited amount of play: I: 59% vs C: 58% (p>0.1)	on asthma control except for limitation in kind of play.<br>Illustrated as no effect but hatched to indicate inconsistent
		Urgent healthcare services<br>Urgent events: No significant between group difference in:<br>• % with ≥1 ED visit/12m: I:12%, vs C: 10% (p > 0.1)<br>• % with ≥1 unscheduled visit/12m: I: 34% vs C: 37% (p >0.1)	No significant effect of intervention in ED visits and unscheduled visits.<br>Illustrated as consistently no effect
Perry, 2018[31]<br>cRCT FU: 6 m<br>USA, Rural minority<br>19 schools: 363 children, Age: 7-14 (mean:9.6)<br><br>Intervention: Telemedicine asthma education<br><br>RoB: Unclear	Intervention characteristics<br>• Based on NAEPP guideline<br>• Tailored to individual and rural population<br>Outer setting<br>• Parents attended session<br>• Letter to HCP 3 monthly<br>Inner setting<br>• Educational session for school nurse<br>• [X] Delivered outside of school hours<br>Individual characteristics<br>• Asthma knowledge and self-efficacy<br>Process<br>• Fidelity: Implemented as planned	School absenteeism<br>Not reported	
		*Asthma control<br>Symptom free days. No significant between-group difference in Symptom free days in last 2w Mean (SD): I: 8.8 (5.1) vs C: 9.4 (5.1), p=0.55	No significant effect of intervention on asthma control<br>Illustrated as no effect
		Urgent healthcare services<br>Not reported	
Persaud, 1996[27]<br>RCT FU: 20wks<br>USA, Urban minority<br>10 schools: 36 children<br>Age: 8-12<br><br>Intervention: Asthma Self-Management<br><br>RoB: High	Intervention characteristics<br>• Based on US guideline<br>• School nurses involved in development<br>• Tailored to urban disadvantaged population<br>Outer setting<br>• [X] parental involvement<br>• Practice accepted insured and non-insured<br>• Letter to HCP<br>Inner setting<br>• Feedback and interview with school staff<br>• Delivered during school hours	School absenteeism<br>School days missed. No significant between group difference in:<br>Days absent/20w mean (SD) I:6.4 (4.6) vs C: 7.6 (5.3), p= NS	No significant effect of intervention on school absenteeism<br>Illustrated as no effect
		Asthma control<br>Not reported	
		Urgent healthcare services<br>ED visits. No significant between-group difference in:<br>• ED visits/20w. Adj mean (SD): I: 0.27 (0.57) vs C: 1.0 (1.2), p=NS<br>Significant between-group difference in proportion of children with ED visits I: 22% vs C: 50%, p<0.05	Adjusted mean was prioritised over proportion in measurement for the outcome. No effect of intervention on ED visits<br>Illustrated as no effect

	Individual characteristics<br>• Asthma knowledge and children asthma attitude<br>Process<br>• Role-play		
Velsor-Friedrich, 2005[28]<br>Non-randomised study<br>FU: 12 m<br>USA, Urban, minority<br>4 schools: 52 children<br>Age: 8-13 (Mean:10.8)<br><br>Intervention: Open Airway for School<br><br>RoB: High	Intervention characteristics<br>• Orem's Self-Care deficit theory<br>• Based on US guideline<br>• Individually tailored by school nurse<br>Outer setting<br>• [x] parental involvement<br>• Coordination with school-based clinic physician<br>Inner setting<br>• Delivered during school hours<br>Individual characteristics<br>• Measured care abilities and self-care practices<br>Process<br>• Group discussion, stories, games, role-play	School absenteeism<br>School days missed. No significant between group difference in:<br>Days absent/yr mean (SD): I: 9.0 vs C: 14.4, p=NS	No significant effect of intervention on missed school days<br>Illustrated as no effect
		Asthma control<br>Days with symptoms. No significant between-group difference in:<br>• % with ≥1 day of symptoms in past 2w: I: 50% vs C: 54%, p=NS	No significant effect of intervention on asthma control<br>Illustrated as no effect
		Urgent healthcare services<br>Urgent doctor visits. No significant between-group difference in:<br>% with ≥1 day of symptoms in past 2w: I: 14% vs C: post:20% , p=NS	No significant effect of intervention on urgent care visits<br>Illustrated as no effect

* indicates primary outcome of the study

Abbreviations: adjOR=adjusted Odds Ratio, adjRR=adjusted Risk Ratio, ACT= Asthma Control Test, ACQ= Asthma Control Questionnaire, C= Control, CAN= Control Asma en Niήos CHSA =Children's Health Survey for Asthma (parent version) and CHSA-C (child version). cRCT = cluster Randomised Controlled Trial, CI= Confidence Interval, ED= Emergency Department, FU= Follow up, HCP= HealthCare Professional, RCT = Randomised Controlled Trial, I=Intervention, RoB = Risk of Bias, RR=Risk Ratio, MCID: Minimal Clinically Importance Difference, NAEPP= National Asthma Education and Prevention Programme, NS=Not Significant, SABA= Short Acting Beta Antagonist, SE= Standard Error, SD= Standard Deviation, m=month, PE= Physical Education, PBL=Problem Based Learning, vs=versus, UK = United Kingdom, USA/US =United States of America, w= week, yr=year

Supplement Table 2: Risk of bias table using the Cochrane EPOC guideline

Author, year,	Random sequence generation	Allocation concealment	Baseline outcome measurements similar	Baseline character similar	Incomplete outcome data	Knowledge of allocated intervention adequately prevented	Protection against contamination	Selective reporting	Other risk of bias	Overall risk of bias
Bartholomew, 2006	Unclear	Low	Unclear	Unclear	Low	Low	Low	Low	Low	Unclear
Cicutto, 2005	Low	Low	Low	Low	Low	Low	Low	Low	Low	Low risk
Cicutto, 2013	Low	Low	Low	Low	Low	Low	Low	Low	Low	Low risk
Clark, 2004	Low	Low	Low	Low	High	Low	Low	Low	Low	High risk
Clark, 2005	Unclear	Low	Unclear	Unclear	Low	Low	Low	Low	Low	Unclear
Clark, 2010	Low	Low	Low	Low	Unclear	Low	Low	Low	Low	Unclear
Gerald, 2006	Unclear	Low	Unclear	Low	Unclear	Low	Low	Low	Low	Unclear
Horner, 2016	Unclear	Low	Low	Low	Low	Low	Low	Low	Low	Unclear
Isik, 2020	Low	Unclear	Low	Low	Low	Low	High	Low	Low	High risk
Levy, 2006	Unclear	High	Low	Low	High	Low	Low	Low	Low	High risk
Magzamen, 2008	High	High	High	High	Unclear	Low	Low	Low	Low	High risk
Marsland, 2018	Unclear	Low	Low	Low	Low	High	Unclear	Low	Low	High risk

Author, year,	Random sequence generation	Allocation concealment	Baseline outcome measurements similar	Baseline character similar	Incomplete outcome data	Knowledge of allocated intervention adequately prevented	Protection against contamination	Selective reporting	Other risk of bias	Overall risk of bias
McCAnn, 2006	Unclear	Low	Low	Low	Unclear	Low	Low	Low	Low	Unclear
McGhan, 2003	Unclear	Low	Low	Low	Unclear	Low	Low	Low	Low	Unclear
McGhan, 2010	Low	Low	Low	Low	Low	Low	Low	Low	Low	Low risk
Prena-Crespo, 2016	Low	Low	Low	Low	Low	Low	Low	Low	Low	Low risk
Perry, 2018	Unclear	Low	Low	Low	Unclear	Low	Low	Low	Low	Unclear
Persaud, 1996	Unclear	Low	Low	Low	High	Low	Unclear	Low	Low	High risk
Simoneau, 2020	High	High	High	High	Low	High	High	Low	Low	High risk
Spencer, 2000	High	High	High	High	High	Low	Low	Low	Low	High risk
Suwannakeeree, 2016	High	High	High	High	Unclear	Low	Low	Low	Low	High risk
Szetler, 2019	High	Low	High	High	Unclear	Low	Low	Low	Low	High risk
Velsor-Friedrich, 2005	High	Low	Low	High	Unclear	Low	Low	Low	Low	High risk

Supplementary Table 3: The domains and subdomains of Consolidated Framework of Implementation Research (CFIR)

Domains in CFIR	Subdomains of CFIR
Intervention characteristics<br>• Features of an intervention that might influence implementation	Theory-driven
	Evidence-based (e.g. based on GINA, CPGs)
	Stakeholder involvement in development (involvement of participants, parents, school staffs in development of intervention)
	Tailored e.g. cultural beliefs and values
Outer Setting<br>• Features of external context and environment that might influence implementation	Parental involvement in intervention (education/information of intervention to parents; not including provision of questionnaire or letter to/from healthcare provider)<br>Substantial parental involvement: parents attended session(s) or received the intervention via active communication e.g. phone call/video call.<br>Minimal parental involvement: parent only received intervention via passive communication e.g. provision of pamphlets/letter via children.<br>No parental involvement: parent did not receive educational materials on the intervention.
	Access to asthma care (yes if more than 80% has universal care/health benefit/insurance)
	Coordination with child's healthcare provider
Inner Setting<br>• Features of implementing organization that might influence implementation	School participation (involvement of school staff e.g. administration, teachers, school nurse included if they are part of school staff/assigned to a school)
	Done during school hours.
Characteristics of Individuals<br>• Features that might influence implementation	Change in individual character e.g. improvement of knowledge, skill or practice.
Process<br>• Strategies or tactics that might influence implementation	Process evaluation: Fidelity (adherence to core components of the intervention)
	Child satisfaction e.g. fun interactive sessions

Footnote: CPG= Clinical practice guidelines, GINA = Global Initiative for Asthma,

Supplement Table 4: Details of CFIR sub-domains for included studies

Studies with positive outcome(s)														
CFIR domains and subdomains	Cicutto 2013	Cicutto, 2005	Clark 2004	Clark 2005	Isik, 2020	Levy 2006	Magzamen 2008	Marsland 2019		Simoneau 2020	Spencer 2000	Suwannakeree 2016	Szetler 2019	Total criteria met
								ICC	OAS					
Intervention characteristics														
Theory-driven	Social cognitive	Social cognitive and self-regulation		Social cognitive	Orem self-care			Life stress model						5
Evidence-based (e.g GINA, CPGs)	Canadian	Canadian		GINA and NAEPP	National guideline	NHLBI		**No**	NHLBI			Used tailored asthma action plan	NAEPP	8
Stakeholder involvement in development (children, parent, school staffs)	Schools and local agencies	HCPs, asthma experts and families			School nurses		Staff, school and peer educator	HCPs and school nurse		Based on opinion form school nurse, parents and HCPs			School nurses, school staff.	7
Tailored e.g. individual, cultural beliefs and values	Local schools and l agencies developed and implemented	Tailored at individual level e.g. health access barrier including cultural beliefs	Adapted to local needs	Adapted to local needs	Tailored to the children's conditions.		Developed by local school	**No**				Tailored to challenges that the child was facing	Developed by experienced bilingual asthma counsellors.	8

Studies with positive outcome(s)														
CFIR domains and subdomains	Cicutto 2013	Cicutto, 2005	Clark 2004	Clark 2005	Isik, 2020	Levy 2006	Magzamen 2008	Marsland 2019		Simoneau 2020	Spencer, 2000	Suwannakeree 2016	Szetler 2019	Total criteria met
								ICC	OAS					
Outer setting														
Substantial parental involvement (received intervention directly from deliverer of intervention)	Parents attended showcase + coordination of care	Parents attended session at school + homework	School fair parents and assignment and homework	School fair for parents with Q& A session for adults		Parents involved in care coordination and received follow-up calls weekly		Parents attended of the sessions, telephone calls or text messages		Parents attended session at school	Parents attended session at school and letter	Parent attended session at school	Parents attended sessions	10
Minimal parental involvement (received intervention indirectly)					Parent received information sheet each session		Customised letter to parents with specific needs and goals		Bring home message to parents					3
Access to asthma care (yes if < 80% has access)	UHC	UHC			Not assessed	80% were insured		**Not assessed**				Asthma care provided for each child in the study	85% were insured	5

Studies with positive outcome(s)														
CFIR domains and subdomains	Cicutto 2013	Cicutto, 2005	Clark 2004	Clark 2005	Isik, 2020	Levy 2006	Magzamen 2008	Marsland 2019		Simoneau 2020	Spencer, 2000	Suwannakeree 2016	Szetler 2019	Total criteria met
								ICC	OAS					
Outer setting														
Coordination with child's healthcare provider	Letter sent to family to provide to HCP	Communicated/coordinated with local specialty care asthma clinic	Not successful (unable to contact most HCP)	Not achieved	No	Contacted HCP		**No**		Communicated with child HCP		Detail of the spirometry results and other information to doctor	Letter to HCP and requested PAAP at 3 intervals	6
Inner setting														
School participation (involvement of school staffs)	Session for broader school community, and resource kit for school	No, only Permission to conduct and advertisement within school community	Session for principals and counsellors	Session for school principals and counsellors	No	Service training of school staff yearly, weekly dialogue with school if needed	**Laison staff worked with health educator**	Teachers were involvement in design, recruitment and engagement of children		Delivered by school nurse		Teachers were trained to assess the child aid their asthma management	School nurse were part of the school staff who conducted the study and provided care	8
Done during school hours	Lunch time	Mostly during lunches /class time			During school hours		Lunch time and lunch provided	During school days				During school hours	Done during class time	7

Studies with positive outcome(s)														
CFIR domains and subdomains	Cicutto 2013	Cicutto, 2005	Clark 2004	Clark 2005	Isik, 2020	Levy 2006	Magzamen 2008	Marsland2019		Simoneau 2020	Spencer, 2000	Suwannakeree 2016	Szetler 2019	Total criteria met
								ICC	OAS					
**Individual characteristics**														
Measurement of individual character e.g. knowledge, skill or practice	Inhaler technique skills score	Self-efficacy score	Parents management index	Parent management index of items	Asthma management test	Asthma knowledge	Asthma management behaviour - PEFR and spacer use	Asthma self-management score		Inhaler technique delivered and assessed	Management of asthma symptoms	Asthma management behaviours	Inhaler technique score	12
**Process**														
Process evaluation – adherence to core components (fidelity)	Implemented as designed	Implemented as designed	No, one key element was not successful conducted (HCP coordination)	Delivered all core components except for coordination with HCP	Implemented as planned (Child attended a make-up session if he/she missed any)			Implemented as planned		**Only 25% implemented up to 3 core elements**			School nurses involved in the program all throughout implementation of the study.	5

Studies with positive outcome(s)														
CFIR domains and subdomains	Cicutto 2013	Cicutto, 2005	Clark 2004	Clark 2005	Isik, 2020	Levy 2006	Magzamen 2008	Marsland2019		Simoneau 2020	Spencer, 2000	Suwannakeree 2016	Szetler 2019	Total criteria met
								ICC	OAS					
Process														
Child satisfaction e.g. fun interactive sessions	Interactive using games, puppetry, arts, skills and homework	Interactive includes puppetry, games, role playing, model building		Games, role-play, artistic activities	PBL, storytelling, colouring/drawings, hands-on experiences etc		Skits, games, videos, role play	15 minutes games/ activities in each session	Games and stories			**No**	Interactive teaching sessions, engagement with the students and their families.	8
Total sub-domains met in individual study	12	11	4	7	9	6	6	8	6	5	2	8	11	2-12

Footnote: blue boxes are responses from authors via email, orange text are responses that did not meet the criteria, blank boxes = no information from the paper and no response from author.

Abbreviation: HCP = Healthcare professionals, ICC=I Can Cope, NAEPP= National Asthma Education and prevention programme guideline, NHLI=National Health and Lung Institution, OAS= Open Airway for School, PBL= problem based learning, UHC= Universal Health Coverage

Studies with no effect(s)													
CFIR domains and subdomains	Bartholomew 2006	Clark 2010		Gerald 2006	Horner 2016	McCann 2000	McGhan 2003	McGhan 2010	Praena-Crespo 2016	Perry 2018	Persaud 1996	Velsor-Friedrich 2005	Total criteria met
		OAS	OAS plus										
Intervention characteristics													
Theory-driven	Social cognitive				Bruhn's theoretical Model		Social Cognitive	Social cognitive				Orem's Self-Care Deficit	5
Evidence-based (e.g based on GINA, CPGs)	NAEPP			Used of tailored written asthma action plan	US guideline	National asthma guideline	Canadian	Canadian	**No, expert consensus**	NAEPP	US Department of Health and Human Services	US Departmental of Health	9
Stakeholder involvement in development (e.g, children parents, school staffs)			Children developed materials		Parents involved	School staff involved in writing school policy			Developed jointly between school, teachers and HCP		Feedback and input held with school nurses		5
Tailored e.g. individual, cultural beliefs and values	Tailored to language and health literacy	Adapted from OAS for middle school student of African American and in urban setting		Individually tailored asthma action plan for each child	Developed to the experience of families in rural areas	Study developed based on a local evidence			Tailored to Spanish children	Tailored to individual and rural population	Developed on experience with urban disadvantaged population	Individually tailored by nurse	9

Studies with no effect(s)													
CFIR domains and subdomains	Bartholomew 2006	Clark 2010		Gerald 2006	Horner 2016	McCann 2000	McGhan 2003	McGhan 2010	Praena-Crespo 2016	Perry 2018	Persaud 1996	Velsor-Friedrich 2005	Total criteria met
		OAS	OAS plus										
Outer setting													
Substantial parental involvement (received intervention directly from deliverer of intervention)				Parent attended session at school	Group presentation to parents and booklets		Parents attended session at school	Parent attended session at school	**No**	Telemedicine with parents	**No**	**No**	5
Minimal parental involvement (received intervention indirectly)	Parents received action plans and video	Take home assignments and material for parents											2
Access to asthma care (yes if more than 80% has access)				All children received asthma medication	92% insured				UHC		Yes- resident practice clinic, accepted Medicaid, insured, uninsured		4
Coordination with child's healthcare provider	Tailored letters and training videos to HCP			Coordinate care with school nurse and HCP	**No**		Letter to HCP and contacted HCP when needed	Letter to HCP and contacted HCP when needed	Forms to coordinate asthma care with HCP	Letter to HCP 3 monthly	Letter to HCP regarding child's condition	Develop plan for each student with the school-based clinic HCP	8

Studies with no effect(s)													
**CFIR domains and subdomains**	Bartholomew 2006	Clark 2010		Gerald 2006	Horner 2016	McCann 2000	McGhan 2003	McGhan 2010	Praena-Crespo 2016	Perry 2018	Persaud 1996	Velsor-Friedrich 2005	Total criteria met
		OAS	OAS plus										
Inner setting													
School participation (involvement of school staffs e.g. education session)	School action committee to improve school enviroment	School staff helped in recruitment, tracking student and scheduling intervention		Session for school faculty and staff	No	Session for teachers	Session for teachers	Session for teachers	PE teachers delivered intervention	Session for school nurse (part of school staff)	Ongoing feedback and post study unstructured interview		8
Done during school hours		School hours		PE period	Lunch time				PE period	**No, weekends or nights**	School time	School hours	6
Individual characteristics													
Measurement of individual character e.g. knowledge, skill or practice	Self-efficacy	Asthma related self-regulation behaviour, Management practices scale of children and parents		Knowledge score	Asthma management-parents and child	Children knowledge of asthma, self-confidence and self-esteem	Self-efficacy scale, management behaviour	Medication on use and management behaviour	Newcastle asthma knowledge questionnaire	Parents asthma knowledge, parents and child self-efficacy	Parent and children asthma knowledge, child asthma attitude	Measures of care abilities and self-care practices	11

Studies with no effects														
CFIR domains and subdomains	Bartholomew 2006	Clark 2010		Gerald 2006	Horner 2016	McCann 2000	McGhan 2003	McGhan 2010	Praena-Crespo 2016	Perry 2018	Persaud 1996	Velsor-Friedrich 2005	Total criteria met
		OAS	OAS plus										
Process													
Process evaluation – adherence to core components				**No, program was cut short due to multiple problems faced**	Intervention fidelity was measured				Implemented as planned, great number of participants	Adhered to all components			3
Child satisfaction e.g. fun interactive sessions	Interactive computer program		Videos, plays, games, demonstration,		Vignettes and problem solving	Role play	Puppetry, games, role play, model building,	Interactive using games, videos, role-play	Video, and slide presentation		Role play	Group discussion, stories, games & role-play	9
Total sub-domains met in individual study	8	4	6	8	10	6	7	7	9	7	9	7	4-10

Footnote: blue boxes are responses from authors via email, red text are responses that did not meet the criteria, blank boxes = no information from the paper and no response from author.

Abbreviation: HCP = Healthcare professionals, NAEPP= National Asthma Education and prevention programme guideline, NHLBI = National Heart Lung Blood Institute, PBL= problem-based learning, UHC= Universal Health Coverage

Supplementary Table 5: Search terms and databases used for the systematic review

Medline (Ovid)
1. exp Asthma/
2. asthma$.mp.
3. (antiasthma$ or anti-asthma$).mp.
4. Respiratory Sounds/
5. wheez$.mp.
6. Bronchial Spasm/
7. bronchospas$.mp.
8. (bronch$ adj3 spasm$).mp.
9. bronchoconstrict$.mp.
10. exp Bronchoconstriction/
11. (bronch$ adj3 constrict$).mp.
12. Bronchial Hyperreactivity/
13. Respiratory Hypersensitivity/
14. ((bronchial$ or respiratory or airway$ or lung$) adj3 (hypersensitiv$ or hyperreactiv$ or allerg$ or insufficiency)).mp.
15. ((dust or mite$) adj3 (allerg$ or hypersensitiv$)).mp.
16. or/1-15
17. (randomized or randomised).ab,ti.
18. placebo.ab,ti.
19. dt.fs.
20. randomly.ab,ti.
21. trial.ab,ti.
22. groups,ab.ti.
23. exp Clinical Trial/
24. or/17-23
25. Animals/
26. Humans/
27. 25 not (25 and 26)
28. 24 not 27
29. exp Schools/
30. exp School Health Services/
31. exp School Nursing/
32. school*.ab,ti.
33. academ*.ab,ti.
34. colleg*.ab,ti.
35. lesson*.ab,ti.
36. pupil*.ab,ti.
37. 29 or 30 or 31 or 32 or 33 or 34 or 35 or 36
38. exp Self Care/
39. exp Health Education/

40. exp Case Management/
41. exp Patient Education as Topic/
42. educat*.ab,ti.
43. manag*.ab,ti.
44. self-car*.ab,ti.
45. (self adj3 car*).mp.
46. train*.ab,ti.
47. instruct*.ab,ti.
48. teach*.ab,ti.
49. patient-cent*.ab,ti.
50. (patient adj3 cent*).mp.
51. exp Patient-Centered Care/
52. patient-focus.ab,ti.
53. (patient adj3 focus*).mp.
54. coach*.ab,ti.
55. skill*.ab,ti.
56. (knowledge adj3 develop).mp.
57. tutor*.ab,ti.
58. 38 or 39 or 40 or 41 or 42 or 43 or 44 or 45 or 46 or 47 or 48 or 49 or 50 or 51 or 52 or 53 or 54 or 55 or 56 or 57
59. 16 and 28 and 37 and 58
60. limit 59 to yr="2017 -Current"

Embase(Ovid)

1. 'Schools'.mp.
2. 'Asthma'.mp.
3. 'School Health Services'.mp.
4. 'school'.mp.
5. 'School Nursing'.mp.
6. 'Academy'.mp.
7. 'Academic'.mp.
8. 'Academies'.mp.
9. 'college'.mp.
10. 'Colleges'.mp.
11. 'lesson'.mp.
12. 'Lessons'.mp.
13. 'pupil'.mp.
14. 'Pupils'.mp.
15. 1 or 3 or 4 or 5 or 6 or 7 or 8 or 9 or 10 or 11 or 12 or 13 or 14
16. 'Self Care'.mp.
17. 'Health Education'.mp.
18. 'Case Management'.mp.
19. 'Patient Education'.mp.

20. 'Educate'.mp.
21. 'Education'.mp.
22. 'Educator'.mp.
23. 'Manage'.mp.
24. 'Management'.mp.
25. 'self-care'.mp.
26. 'train'.mp.
27. 'Training'.mp.
28. 'trainer'.mp.
29. 'Instruct'.mp.
30. 'Instructor'.mp.
31. 'Instruction'.mp.
32. 'teach'.mp.
33. 'Teacher'.mp.
34. 'patient-center'.mp.
35. 'patient center'.mp.
36. 'Patient-Centered Care'.mp.
37. 'patient-focus'.mp.
38. 'patient focus'.mp.
39. 'Coach'.mp.
40. 'skill'.mp.
41. 'Skills'.mp.
42. 'knowledge develop'.mp.
43. 'Tutor'.mp.
44. 16 or 17 or 18 or 19 or 20 or 21 or 22 or 23 or 24 or 25 or 26 or 27 or 28 or 29 or 30 or 31 or 32 or 33 or 34 or 35 or 36 or 37 or 38 or 39 or 40 or 41 or 42 or 43
45. 2 and 15 and 44
46. limit 45 to yr="2017 -Current"
47. limit 46 to human

AMED (Ovid)

1. exp Asthma/
2. exp Schools/
3. asthma*.mp.
4. 1 or 3
5. exp School health services/
6. School Nursing.mp.
7. (school* or academ* or colleg* or lesson* or pupil*).mp.
8. 2 or 5 or 6 or 7
9. (educat* or manag* or self-car* or train* or instruct* or teach* or patient-cent or coach* or skill* or tutor*).mp.

10. ((self adj1 car*) or (patient adj1 cent*) or (patient adj1 focus*) or (knowledge adj1 develop*)).mp.
11. exp Self care/
12. exp Health education/
13. "Case management".mp.
14. exp Patient education/
15. exp patient centered care/
16. 9 or 10 or 11 or 12 or 13 or 14 or 15
17. 4 and 8 and 16
18. limit 17 to yr="2017 -Current

PscyhINFO

1. exp Asthma/
2. asthma$.mp.
3. (antiasthma$ or anti-asthma$).mp.
4. Respiratory Sounds/
5. wheez$.mp.
6. Bronchial Spasm/
7. bronchospas$.mp.
8. (bronch$ adj3 spasm$).mp.
9. bronchoconstrict$.mp.
10. exp Bronchoconstriction/
11. (bronch$ adj3 constrict$).mp.
12. Bronchial Hyperreactivity/
13. Respiratory Hypersensitivity/
14. ((bronchial$ or respiratory or airway$ or lung$) adj3 (hypersensitiv$ or hyperreactiv$ or allerg$ or insufficiency)).mp.
15. ((dust or mite$) adj3 (allerg$ or hypersensitiv$)).mp.
16. or/1-15
17. exp Schools/
18. exp School Health Services/
19. exp School Nursing/
20. school*.ab,ti.
21. academ*.ab,ti.
22. colleg*.ab,ti.
23. lesson*.ab,ti.
24. pupil*.ab,ti.
25. 17 or 18 or 19 or 20 or 21 or 22 or 23 or 24
26. exp Self Care/
27. exp Health Education/
28. exp Case Management/
29. exp Patient Education as Topic/

30. educat*.ab,ti.
31. manag*.ab,ti.
32. self-car*.ab,ti.
33. (self adj3 car*).mp.
34. train*.ab,ti.
35. instruct*.ab,ti.
36. teach*.ab,ti.
37. patient-cent*.ab,ti.
38. (patient adj3 cent*).mp.
39. exp Patient-Centered Care/
40. patient-focus.ab,ti.
41. (patient adj3 focus*).mp.
42. coach*.ab,ti.
43. skill*.ab,ti.
44. (knowledge adj3 develop).mp.
45. tutor*.ab,ti.
46. 26 or 27 or 28 or 29 or 30 or 31 or 32 or 33 or 34 or 35 or 36 or 37 or 38 or 39 or 40 or 41 or 42 or 43 or 44 or 45
47. 16 and 25 and 46
48. limit 47 to yr="2017 -Current"

CINAHL –PLUS (Ebscohost)	
S1	asthma*
S2	(MH "Asthma+")
S3	(MH "Schools+")
S4	(MH "School Health Services+")
S5	(MH "Schools, Nursing+")
S6	"school*"
S7	"academ*"
S8	"colleg*"
S9	"lesson*"
S10	"pupil*"
S11	S3 OR S4 OR S5 OR S6 OR S7 OR S8 OR S9 OR S10
S12	MH "Self Care+" OR MH "Health Education+" OR MH "Case Management+" OR MH "Patient Education+" OR educat* OR manag* OR self-car* OR train* OR instruct* OR teach* OR patient-cent* OR self n1 car*
S13	patient n1 cent* OR MH "Patient-Centred Care+" OR patient-focus OR patient N1 focus* OR coach* OR skill* OR knowledge n1 develop* OR tutor*
S14	S12 OR S13
S15	S1 OR S2
S16	S11 AND S14 AND S15

Cochrane CENTRAL

#1 MeSH descriptor: [Asthma] explode all trees

#2 (asthma*):ti,ab,kw

#3 #1 OR #2

#4 MeSH descriptor: [Schools] explode all trees

#5 MeSH descriptor: [School Health Services] explode all trees

#6 MeSH descriptor: [School Nursing] explode all trees

#7 (school*):ti,ab,kw OR (academ*):ti,ab,kw OR (colleg*):ti,ab,kw OR (lesson*):ti,ab,kw OR (pupil*):ti,ab,kw

#8 #4 OR #5 OR #6 OR #7

#9 (educat*):ti,ab,kw OR (manag*):ti,ab,kw OR (self-car*):ti,ab,kw OR (self NEXT car*):ti,ab,kw OR (train*):ti,ab,kw

#10 (instruct*):ti,ab,kw OR (teach*):ti,ab,kw OR (patient-cent*):ti,ab,kw OR (patient NEXT cent*):ti,ab,kw AND (patient-focus*):ti,ab,kw

#11 (patient NEXT focus*):ti,ab,kw OR (coach*):ti,ab,kw OR (skill*):ti,ab,kw OR (knowledge NEXT develop*):ti,ab,kw OR (tutor*):ti,ab,kw

#12 #9 OR #10 OR #11

#13 MeSH descriptor: [Self Care] explode all trees

#14 MeSH descriptor: [Health Education] explode all trees

#15 MeSH descriptor: [Case Management] explode all trees

#16 MeSH descriptor: [Patient Education as Topic] explode all trees

#17 MeSH descriptor: [Patient-Centered Care] explode all trees

#18 #12 OR #13 OR #14 OR #15 OR #16 OR #17

#19 #3 AND #8 AND #18 with Cochrane Library publication date from Aug 2017 to present (February 2019)

Global Health (Ovid)

1. exp Asthma/
2. asthma$.mp.
3. (antiasthma$ or anti-asthma$).mp.
4. Respiratory Sounds/
5. wheez$.mp.
6. Bronchial Spasm/
7. bronchospas$.mp.
8. (bronch$ adj3 spasm$).mp.
9. bronchoconstrict$.mp.
10. exp Bronchoconstriction/
11. (bronch$ adj3 constrict$).mp.
12. Bronchial Hyperreactivity/
13. Respiratory Hypersensitivity/
14. ((bronchial$ or respiratory or airway$ or lung$) adj3 (hypersensitiv$ or hyperreactiv$ or allerg$ or insufficiency)).mp.
15. ((dust or mite$) adj3 (allerg$ or hypersensitiv$)).mp.
16. or/1-15
17. (randomized or randomised).ab,ti.
18. placebo.ab,ti.
19. dt.fs.
20. randomly.ab,ti.
21. trial.ab,ti.
22. groups,ab.ti.
23. exp Clinical Trial/
24. or/17-23
25. Animals/
26. Humans/
27. 25 not (25 and 26)
28. 24 not 27
29. exp Schools/
30. exp School Health Services/
31. exp School Nursing/
32. school*.ab,ti.
33. academ*.ab,ti.
34. colleg*.ab,ti.
35. lesson*.ab,ti.
36. pupil*.ab,ti.
37. 29 or 30 or 31 or 32 or 33 or 34 or 35 or 36
38. exp Self Care/
39. exp Health Education/
40. exp Case Management/
41. exp Patient Education as Topic/

42. educat*.ab,ti.
43. manag*.ab,ti.
44. self-car*.ab,ti.
45. (self adj3 car*).mp.
46. train*.ab,ti.
47. instruct*.ab,ti.
48. teach*.ab,ti.
49. patient-cent*.ab,ti.
50. (patient adj3 cent*).mp.
51. exp Patient-Centered Care/
52. patient-focus.ab,ti.
53. (patient adj3 focus*).mp.
54. coach*.ab,ti.
55. skill*.ab,ti.
56. (knowledge adj3 develop).mp.
57. tutor*.ab,ti.
58. 38 or 39 or 40 or 41 or 42 or 43 or 44 or 45 or 46 or 47 or 48 or 49 or 50 or 51 or 52 or 53 or 54 or 55 or 56 or 57
59. 16 and 28 and 37 and 58

Supplementary Table 6: Characteristics of excluded studies

Study	Reason for exclusion
Abdel-basset, 2018	Non-experimental study
Al-Aloola, 2017	Not within age range
Allen 2018	Not school-based self-management education intervention
Al-Syehab, 2012	Not within the age range
Al-Syehab, 2012a	Not within the age range
Altherly, 2009	Not within the age range
Berg, 2004	Not within the age range
Bignall, 2015	Not within the age range
Brasler, 2006	Not within the age range
Bruzzese, 2004	Not within the age range
Bruzzese, 2010	Not within the age range
Bruzzese, 2011	Not within the age range
Bruzzesse 2018	Not within age range
Bryant Stephan, 2018	Non-experimental study
Carpenter, 2016	No outcome of interest
Coelhe, 2018	No outcome of interest
Crane, 2014	No outcome of interest
David, 2018	Abstract. No full text. No outcome of interest
Dolins, 2017	Not school-based self-management education intervention
Dore-Stites, 2007	Thesis
Dutlova, 2019	Non-experimental study
Eakin, 2018	Not within age range
Elliot, 2019	Abstract. No full text.
Engelke, 2013	No outcome of interest
Evans, 2001	No outcome of interest
Francisco, 2017	Not within age range
Gary, 2017	Abstract. No full text. No outcome of interest.
Gerald, 2009	Not school-based self-management education intervention (intervention was supervised asthma therapy at school)
Greer. M. 2017	No outcome of interest
Halterman, 2018	Not school-based self-management education intervention (intervention was supervised asthma therapy at school)
Haris. 2018.	Abstract. Not full text. No outcome of interest.
Henry, 2004	Not within the age range
Hogan, M. 2017.	Non-experimental study
Holley. 2018.	Not within age range
Horner, 2004	Not school-based self-management education intervention (Intervention were in combination with individualised home-based family education visit)
Horner, 2008	No outcome of interest
Horner, 2014	Not school-based self-management education intervention (Intervention were in combination with individualised home-based family education visit)
Horner, 2018.	Not school-based self-management education intervention
Howell, 2005	Thesis.

Jackson, 2006	No outcome of interest
Jones, 2017	Not within age range
Joseph, 2010	Not within the age range
Joseph, 2013	Not within the age range
Kakumanu, 2018	Non-experimental study
Khan, 2014	Not school-based self-management education intervention
Kintner, 2012	Not within the age range
Kouba, 2012	Not within the age range
Langenfeld, 2010	No outcome of interest
Lee, 2011	No outcome of interest
Mc-Cabe, 2020	Non-experimental study
McClure, 2018.	No outcome of interest
Mickel, 2016	No outcome of interest
Monforte, 2011	Abstract. Only publish article published in conference. No full paper found.
Mosnaim, 2011	No outcome of interest
Mujuru, 2011	No outcome of interest
Naman, 2018.	No outcome of interest
NCT. Telemedicine Enhanced Asthma Management - Uniting Providers	Not school-based self-management education intervention
Newman, 2014	Not school-based self-management education intervention
Patterson, 2005	No outcome of interest
Peers. 2017	Abstract. No full text. No outcome of interest
Perry, 2019	Non-experimental study
Phipatanakul, 2017	Not school-based self-management education intervention
Pike 2011	No outcome of interest
Praena-Crespo, 2010	Not within the age range
Pulcini, 2007	Not within the age range
Radic. 2017	Non-experimental study
Reznik, 2017	Abstract. No full paper found. No outcome of interest
Reznik, 2018.	Abstract. No full paper found. Not within the age range
Richmond, 2011	No outcome of interest
Ross, 2020	Non-experimental study
Shah, 2001	Not within the age range
Srot, 2012	Not within the age range
Syah, 2018; McCallum, 2018	Not within the age range
Trepstra, 2012	Not within age range
Tzeng, 2018	Non-experimental study
Urrutia-Perreira, 2018	Non-experimental study
Volerman, 2017	Abstract. No full paper found. No outcome of interest
Yin, 2017	Not within the age range
